# Effects of Ellagic Acid on Gut Microbiota and Tryptophan Metabolism in Paraquat-Challenged Piglets Subjected to Ampicillin-Induced Microbiota Perturbation

**DOI:** 10.3390/antiox15101356

**Published:** 2026-10-20

**Authors:** Yuankun Deng, Chengming Wang, Yunlong Meng, Yulong Yin, Jing Wang, Qiuping Ye

**Affiliations:** 1 Xiangya School of Nursing, Central South University, Changsha 410013, China; 2 Hunan Provincial Key Laboratory for the Products Quality Regulation of Livestock and Poultry, College of Animal Science and Technology, Hunan Agricultural University, Changsha 410128, China; 3 Department of Animal Science, Shandong Agricultural University, Taian 271017, China; 4 Laboratory of Animal Nutritional Physiology and Metabolic Process, Key Laboratory of Agro-Ecological Processes in Subtropical Region, National Engineering Laboratory for Pollution Control and Waste Utilization in Livestock and Poultry Production, Institute of Subtropical Agriculture, Chinese Academy of Sciences, Changsha 410125, China; 5 School of Nursing, Guiyang Kangyang University, Guiyang 550081, China

**Keywords:** ellagic acid, oxidative stress, gut microbiota, tryptophan metabolism, piglets

## Abstract

Oxidative stress (OS) is closely associated with intestinal dysfunction, gut microbial dysbiosis, and metabolic disturbances in weaned piglets. Ellagic acid (EA) is a dietary polyphenol with antioxidant properties. This study investigated the effects of EA supplementation and ampicillin (AMP)-induced microbial perturbation on OS, gut microbiota, and tryptophan (Trp) metabolism in paraquat (PQ)-challenged piglets. Twenty-four weaned piglets were assigned to four treatments in a 2 × 2 factorial design (±EA, ±AMP), followed by a PQ challenge. Neither EA nor AMP affected growth performance or most serum biochemical parameters. EA significantly decreased malondialdehyde (MDA) in AMP-treated piglets. AMP altered ileal morphology and reshaped the microbial community in both the ileum and colon. Moreover, EA and AMP differentially affected Trp metabolism in serum and intestinal segments; AMP altered kynurenine-pathway and microbiota-associated indole metabolites, with significant EA × AMP interactions for some metabolites. Spearman analysis further revealed distinct associations between gut microbiota and Trp metabolites along the intestinal tract. Collectively, AMP-induced microbial perturbation was accompanied by substantial Trp-metabolism alterations and distinct microbiota–Trp associations, whereas EA modulated MDA levels and selected microbial and metabolic responses under AMP treatment. These observations suggest that EA effects under OS may be associated with gut microbiota-Trp metabolism axis modulation, although causality remains to be established.

## 1. Introduction

Oxidative stress (OS) occurs when reactive oxygen species (ROS) production exceeds antioxidant defenses, disrupting redox homeostasis. Although physiological levels of ROS participate in cellular signaling, excessive ROS can damage lipids, proteins, and DNA and promote inflammation [1,2]. OS is also closely linked to intestinal microbial dysbiosis. An altered intestinal redox environment can affect microbial composition and function, whereas dysbiosis and barrier dysfunction can facilitate microbial product translocation and exacerbate inflammation and oxidative injury [3,4,5,6,7]. These reciprocal interactions identify the gut microbiota as a potential target for nutritional interventions against OS.

Ellagic acid (EA), a dietary polyphenol found in pomegranates, berries, and nuts, exhibits antioxidant and anti-inflammatory properties through mechanisms involving nuclear factor erythroid 2-related factor 2 (Nrf2) and nuclear factor-κB (NF-κB) signaling [8,9]. Its limited intestinal absorption and microbial conversion into bioactive metabolites, including urolithins, suggest that host–microbiota interactions contribute to its biological effects [10,11,12]. EA can also modulate gut microbial composition and metabolic function [13,14]. Of particular interest, microbial tryptophan (Trp) metabolism generates indole derivatives that participate in redox regulation, immune homeostasis, and intestinal barrier maintenance, partly through aryl hydrocarbon receptor (AhR)-associated pathways [15,16,17,18,19,20,21]. Although EA has been associated with changes in the gut microbiota and Trp metabolism [13,21,22,23], their contribution to its protective effects against OS remains unclear.

Our previous study showed that EA alleviated paraquat (PQ)-induced OS and intestinal injury in piglets, with protection associated with Nrf2 signaling [9]. Similar Nrf2-related antioxidant effects have been reported in PQ-exposed A549 cells and diquat-challenged mice [24,25]. PQ undergoes intracellular redox cycling, generating superoxide anions while consuming reducing equivalents such as NADPH [26]. This mechanism, together with our previous findings, supports its use to investigate EA-mediated protection against oxidative injury. To examine potential microbial involvement, ampicillin (AMP) was included as a gut microbiota perturbation. This broad-spectrum β-lactam antibiotic has been shown to alter microbial diversity, community structure, and metabolite profiles in mice [27], providing a rationale for testing whether AMP treatment modifies the response to EA.

We hypothesized that EA-mediated protection partly involves the gut microbiota and microbial Trp metabolism and may therefore be attenuated by AMP-induced microbial perturbation. To test this hypothesis, we employed a 2 × 2 factorial design incorporating EA supplementation and AMP treatment, with all piglets exposed to PQ. By integrating 16S rRNA sequencing, metabolomic analysis, biochemical assays, and intestinal histological assessment, we assessed whether AMP modifies the effects of EA on OS and intestinal injury and examined accompanying microbial and Trp metabolic changes. This study aimed to clarify the potential contribution of microbiota-associated metabolism to the protective effects of EA.

## 2. Materials and Methods

### 2.1. Animals and Experimental Design

**Figure 1:**
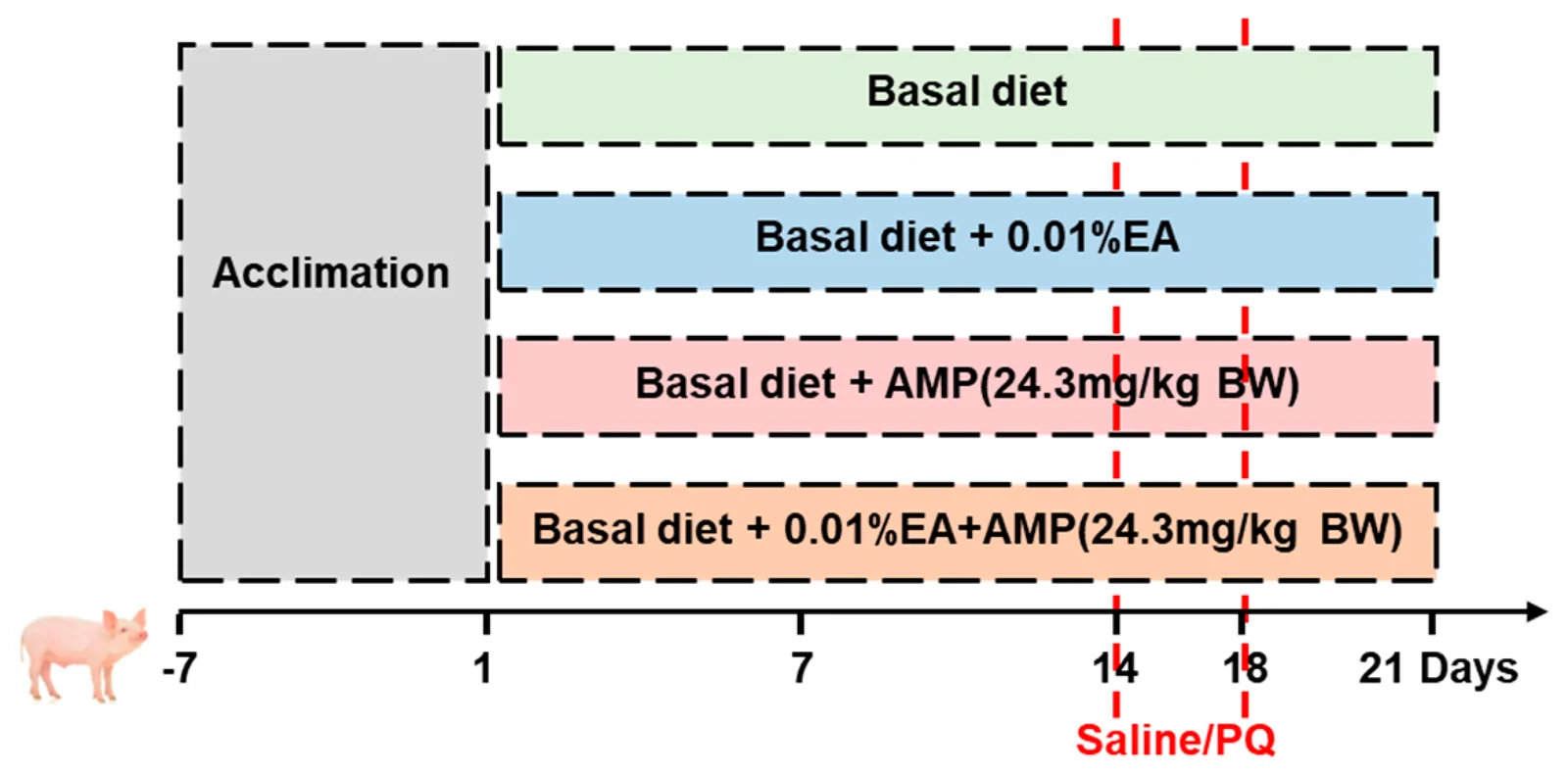
Animal experimental design.

Twenty-four healthy, 21-day-old weaned male piglets (Duroc × Landrace × Yorkshire) with similar initial body weights were obtained from 15 litters. All piglets were housed individually in pens located in the same room, where the ambient temperature was maintained at 22 ± 1 °C and relative humidity at 40–70%. Piglets had free access to drinking water throughout the study. A 7-day acclimation period preceded the 21-day experiment. A 2 × 2 factorial design was used, with dietary EA supplementation (0 or 0.01%) and AMP administration (0 or 24.3 mg/kg body weight) as the two factors. Piglets were randomly assigned to four treatment groups, with six replicates per group and one piglet per replicate: (1) Control (AMP−/EA−); (2) EA (AMP−/EA+); (3) AMP (AMP+/EA−); and (4) AMP + EA (AMP+/EA+). The experimental design and treatment schedule are shown in Figure 1. Throughout the experimental period, piglets in the Control and AMP groups were fed a basal diet, whereas those in the EA and EA + AMP groups were fed the basal diet supplemented with 0.01% EA. Piglets in the EA + AMP and AMP groups received AMP at 24.3 mg/kg body weight, dissolved in 5 mL of water, by oral gavage once daily on days 1–21. Piglets in the Control and EA groups received 5 mL of physiological saline per piglet by oral gavage once daily over the same period. On experimental days 14 and 18, all 24 piglets received intraperitoneal injections of PQ at 4 mg/kg body weight per injection to induce OS. At the end of the experiment, all piglets were euthanized, and blood, intestinal tissues, and other relevant samples were collected for subsequent analyses. The composition and nutrient levels of the basal diet are presented in Table 1. The basal diet met the nutritional requirements recommended by the National Research Council (NRC).

### 2.2. Sample Collection

At the end of the experiment, piglets were euthanized for the collection of blood and intestinal samples. Approximately 10 mL of blood was aseptically collected from the jugular vein, centrifuged at 3000 rpm for 10 min at 4 °C, and the obtained serum was stored at −20 °C until further analysis. A 2 cm segment of the mid-ileum was collected, fixed in 4% paraformaldehyde, and subjected to histomorphology evaluation. Following complete dissection of the abdominal digestive tract, luminal contents were collected from the mid-ileum and mid-colon. The ileal and colonic segments were longitudinally opened along the mesenteric border, gently rinsed with sterile physiological saline, and intestinal mucosal samples were carefully scraped. The collected ileal and colonic mucosa samples were immediately snap-frozen in liquid nitrogen and stored at −80 °C for subsequent molecular analyses.

### 2.3. Growth Performance

Individual body weights were recorded at the beginning and end of the experimental period after overnight fasting. Feed intake was recorded daily throughout the trial, and all measurements were obtained using calibrated electronic scales (±0.01 kg). ADFI (average daily feed intake) = Total feed intake/(Number of animals × Trial period); ADG (average daily gain) = Total weight gain/(Number of animals × Trial period). Organ indices were calculated as organ weight relative to final body weight using the following formula: organ index = organ weight (g)/final body weight (kg).

### 2.4. Blood Parameters Analysis

Serum biochemical parameters were determined spectrophotometrically using an automated biochemical analyzer (BS-200; Mindray, Shenzhen, China). The measured parameters included alanine aminotransferase (ALT), aspartate aminotransferase (AST), alkaline phosphatase (ALP), total protein (TP), albumin (ALB), globulin (GLB), glucose (GLU), blood urea nitrogen (BUN), total cholesterol (TC), triglycerides (TG), high-density lipoprotein cholesterol (HDL-C), and low-density lipoprotein cholesterol (LDL-C).

### 2.5. Determination of Serum Total Antioxidant Capacity (T-AOC) and Malondialdehyde (MDA) Levels

T-AOC (A015-2-1) and MDA (A003-1-2) concentrations were measured using commercial assay kits from Nanjing Jiancheng Bioengineering Institute (Nanjing, China) according to the manufacturer’s instructions.

### 2.6. Determination of Serum Diamine Oxidase (DAO) and D-Lactate (DLA) Levels

DAO (G0134F) and DLA (G0827F) concentrations in serum were measured according to the manufacturer’s instructions contained in the porcine ELISA kits (Suzhou Grace Bio-Tech Co., Ltd., Suzhou, China).

### 2.7. Intestinal Morphology Analysis

An approximately 2-cm ileal segment was collected from a site approximately 10 cm proximal to the ileocecal junction. The tissue was fixed in 4% paraformaldehyde at room temperature for 24 h, dehydrated through a graded ethanol series, cleared, and embedded in paraffin. Sections were cut at a thickness of 3 μm, mounted on glass slides, deparaffinized, rehydrated, and stained with hematoxylin and eosin (H&E). Images were acquired using a microscope equipped with a 20× objective. Histomorphometric analysis was performed by an investigator blinded to treatment allocation using CaseViewer software (version 2.4). One section per animal was analyzed, and villus-crypt units were randomly selected from eligible structures. Eligible units had well-oriented, intact villi extending from the base to the tip and corresponding crypts with clearly identifiable bases. Obliquely sectioned, folded, or mechanically damaged structures were excluded. Villus height (VH) was measured from the villus-crypt junction to the villus tip, and crypt depth (CD) was measured from the same junction to the crypt base. Mean VH and CD were calculated for each animal, and the VH/CD ratio was determined. Each animal was considered an experimental unit for statistical analysis.

### 2.8. Serum-Free Amino Acid Profile Analysis

Serum free amino acid concentrations were determined using an automatic amino acid analyzer (L-8900, Hitachi, Tokyo, Japan). This analyzer operates based on ion-exchange chromatography coupled with ninhydrin colorimetric detection. Briefly, serum samples were deproteinized with 0.8% sulfosalicylic acid solution and incubated at 4 °C for 15 min. After centrifugation at 10,000 rpm for 20 min, the supernatants were collected and filtered through a 0.22 μm membrane filter prior to injection. An amino acid mixed standard solution was used for calibration, and quantification was performed using the external standard method. Calibration curves were constructed with serial dilutions of the standard solution, and standard solutions were run alongside samples in each analytical batch for instrument calibration.

### 2.9. Intestinal Microbiome Analysis

Ileal and colonic contents were collected from 24 piglets, yielding 24 samples from each intestinal segment. Bacterial DNA was extracted using the TIANamp Stool DNA Kit (DP328; TIANGEN; Beijing, China) according to the manufacturer’s instructions. A blank extraction control was included using nuclease-free water instead of intestinal content. The extracted DNA was eluted in 50 μL of elution buffer and stored at −80 °C until PCR amplification. The V3-V4 regions of the bacterial 16S rRNA gene were amplified using primers carrying sample-specific barcodes. Each 25-μL PCR mixture contained 25 ng of template DNA, 12.5 μL of PCR premix, and 2.5 μL each of the forward and reverse primers, with PCR-grade water added to the final volume. The amplification program consisted of initial denaturation at 98 °C for 30 s; 32 cycles of denaturation at 98 °C for 10 s, annealing at 54 °C for 30 s, and extension at 72 °C for 45 s; and a final extension at 72 °C for 10 min. PCR products were examined by electrophoresis on a 2% agarose gel, purified using AMPure XT beads (Beckman Coulter Genomics, Danvers, MA, USA), and quantified using a Qubit fluorometer (Invitrogen, Carlsbad, CA, USA). Amplicon libraries were assessed using an Agilent 2100 Bioanalyzer (Invitrogen, Carlsbad, CA, USA) and a Library Quantification Kit for Illumina (Kapa Biosciences, Woburn, MA, USA). Paired-end sequencing was performed on an Illumina NovaSeq PE250 platform by LC-Bio Technology Co., Ltd. (Hangzhou, China).

Sequence processing, taxonomic annotation, and statistical analyses were performed on the Novogene NovoMagic amplicon analysis cloud platform (https://magic.novogene.com), with the core workflow based on QIIME 2 (version 2023.2). Paired-end reads were demultiplexed according to their sample-specific barcodes, and barcode and primer sequences were removed. Reads were merged using FLASH and quality-filtered using fqtrim (version 0.9.4), retaining sequences with a mean Phred quality score ≥20 and a length ≥ 200 bp. Chimeric sequences were subsequently removed using VSEARCH (version 2.3.4). Amplicon sequence variants (ASVs) were inferred using DADA2. Following quality control, the number of retained sequences ranged from 42,368 to 68,592 per sample, with a minimum of 42,368 sequences. To account for differences in sequencing depth, all samples were rarefied to 42,368 sequences per sample before alpha and beta diversity analyses. Representative ASV sequences were taxonomically classified against the SILVA 16S rRNA gene database (release 138) using the naive Bayes classifier implemented in QIIME 2. Microbial composition, diversity, and differential abundance analyses were performed separately for ileal and colonic samples. Alpha diversity indices, including Shannon, Chao1, and Simpson, were calculated using QIIME 2. Beta diversity was assessed using Bray-Curtis dissimilarity. Group differences in alpha diversity indices and Bray-Curtis dissimilarity values were evaluated using Kruskal-Wallis tests. Beta diversity was evaluated using Bray-Curtis distance matrices, and differences in microbial community structure among treatment groups were tested using permutational multivariate analysis of variance (PERMANOVA) with 999 permutations. Differentially abundant taxa were identified using linear discriminant analysis effect size (LEfSe), with an LDA score threshold of 3.0. *p*-values from multiple comparisons were adjusted using the Benjamini-Hochberg method, and an adjusted *p* < 0.05 was considered statistically significant.

### 2.10. Trp Metabolite Analysis

Ultra-high-performance liquid chromatography coupled with tandem mass spectrometry (UPLC-MS/MS) was employed for the targeted quantitative analysis of 30 tryptophan and its metabolites. Briefly, serum samples (50 μL) and tissue/digestate homogenates (equivalent to 50 mg wet weight) were extracted with 80% aqueous methanol, homogenized/vortexed, diluted with 10% aqueous methanol, and centrifuged at 12,000× *g* for 10 min at 4 °C. The supernatant was diluted with 50% aqueous methanol, spiked with deuterated internal standard (L-tryptophan-d_3_, 10 ppb), filtered through a 0.22-μm PTFE membrane, and analyzed on a Waters ACQUITY UPLC^®^ HSS T3 column (2.1 × 150 mm, 1.8 μm; Waters Corporation, Milford, MA, USA)) coupled to an AB SCIEX QTRAP^®^ 5500 mass spectrometer (SCIEX, Framingham, MA, USA). Chromatographic separation and MS parameters are detailed in Supplementary File S1.

### 2.11. Correlation Analysis

Partial Spearman rank correlations were calculated to assess associations between gut microbial taxa and tryptophan metabolites across all 24 piglets, adjusting for EA and AMP treatments as binary covariates (absent or present). *p*-values were adjusted for multiple testing across all taxon–metabolite pairs using the Benjamini-Hochberg procedure.

### 2.12. Statistical Analysis

The experiment followed a 2 × 2 factorial design with two dietary EA levels (0 and 0.01%) and two AMP treatment levels (0 and 24.3 mg/kg body weight), with six piglets per treatment combination. Each piglet was considered an experimental unit. Two-way analysis of variance (ANOVA) was performed using the general linear model (GLM) procedure of SPSS software (version 20.0; SPSS Inc., Chicago, IL, USA) to evaluate the main effects of EA and AMP and their interaction. When a significant EA × AMP interaction was detected, simple main effects analyses were performed. All data are presented as means ± standard error of the mean (SEM). Statistical significance was defined as *p* < 0.05.

## 3. Results

### 3.1. Effects of EA and AMP on Growth Performance in PQ-Challenged Piglets

The main effects of EA and AMP and their interaction on growth performance and organ indices are presented in Table 2. Neither EA nor AMP significantly affected BW, ADG, ADFI, or the heart, spleen, and kidney indices (*p* > 0.05). EA supplementation tended to increase the liver index (*p* = 0.053). No significant EA × AMP interactions were detected for growth performance or organ indices (*p* > 0.05).

### 3.2. Effects of EA and AMP on Serum Biochemical Parameters in PQ-Challenged Piglets

As shown in Table 3, EA supplementation tended to decrease serum AST (*p* = 0.056) and increase TG (*p* = 0.058) concentrations. AMP treatment increased serum ALB (*p* < 0.05), decreased TG (*p* < 0.05), and tended to increase TP (*p* = 0.053). No significant EA × AMP interactions were detected for the measured biochemical parameters (*p* > 0.05), although the interactions for ALB (*p* = 0.055) and TP (*p* = 0.063) approached significance.

### 3.3. Effects of EA and AMP on Serum Free Amino Acid Profiles in PQ-Challenged Piglets

As shown in Table 4, EA supplementation significantly increased serum Ala and Pro concentrations (*p* < 0.05) and tended to increase Met (*p* = 0.057). AMP treatment did not significantly affect the concentrations of most serum free amino acids (*p* > 0.05), although it tended to increase Cys concentrations (*p* = 0.056) and decrease Car concentrations (*p* = 0.052). Significant EA × AMP interactions were observed for serum Cit and Cys concentrations (*p* < 0.05). Simple effects analysis showed that AMP significantly decreased Cit levels and increased Cys levels in the presence of EA, and EA significantly decreased Cit levels and increased Cys levels in the presence of AMP (*p* < 0.05).

### 3.4. Effects of EA and AMP on Antioxidant Status

**Figure 2:**
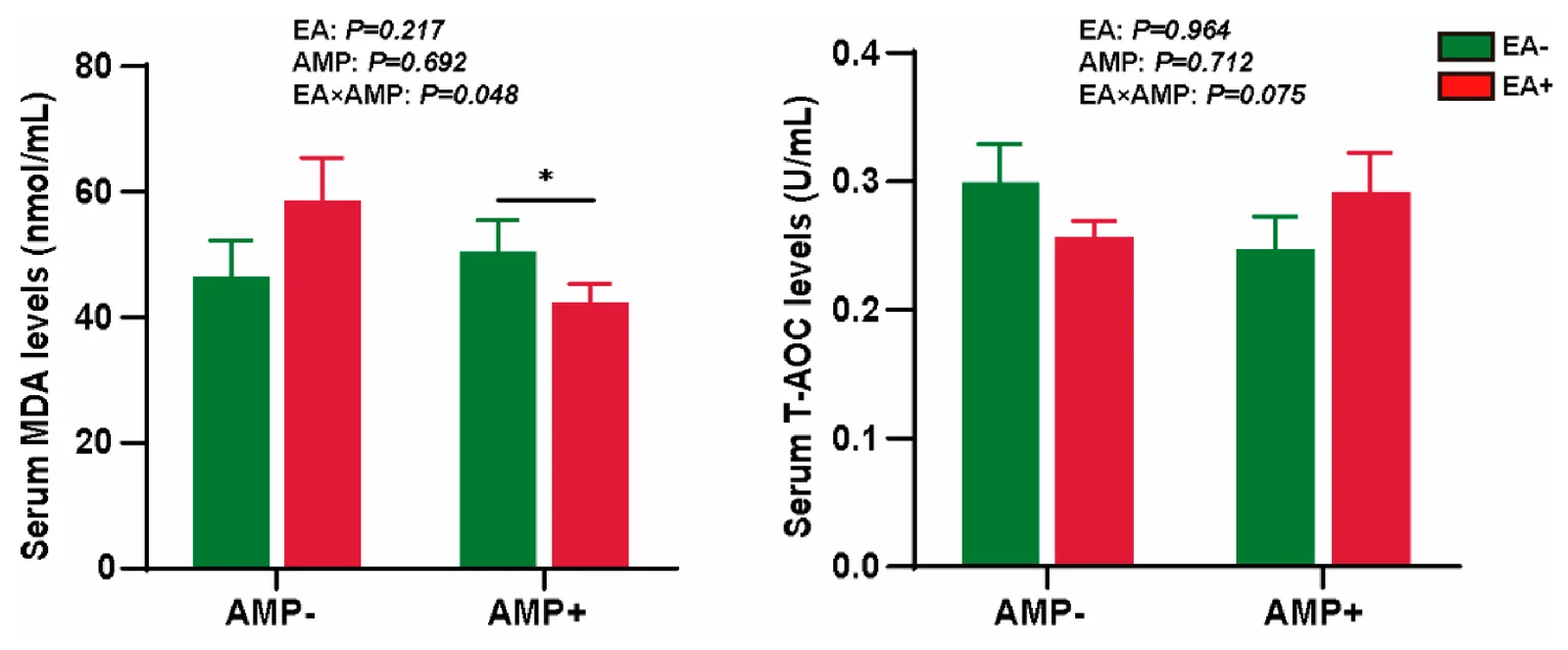
Effects of EA and AMP supplementation on serum MDA and T-AOC levels. Data are shown as mean ± SEM, *n* = 6 for each group. * *p <* 0.05.

The effects of EA and AMP on OS-related parameters are presented in Figure 2. Neither EA nor AMP exerted a significant main effect on serum MDA or T-AOC (*p* > 0.05). A significant EA × AMP interaction was detected for MDA (*p* < 0.05). Simple effects analysis showed that EA supplementation significantly decreased MDA levels in the presence of AMP (*p* < 0.05). No significant EA × AMP interaction was observed for T-AOC (*p* > 0.05).

### 3.5. Effects of EA and AMP on Intestinal Barrier Function and Intestinal Morphology in PQ-Challenged Piglets

**Figure 3:**
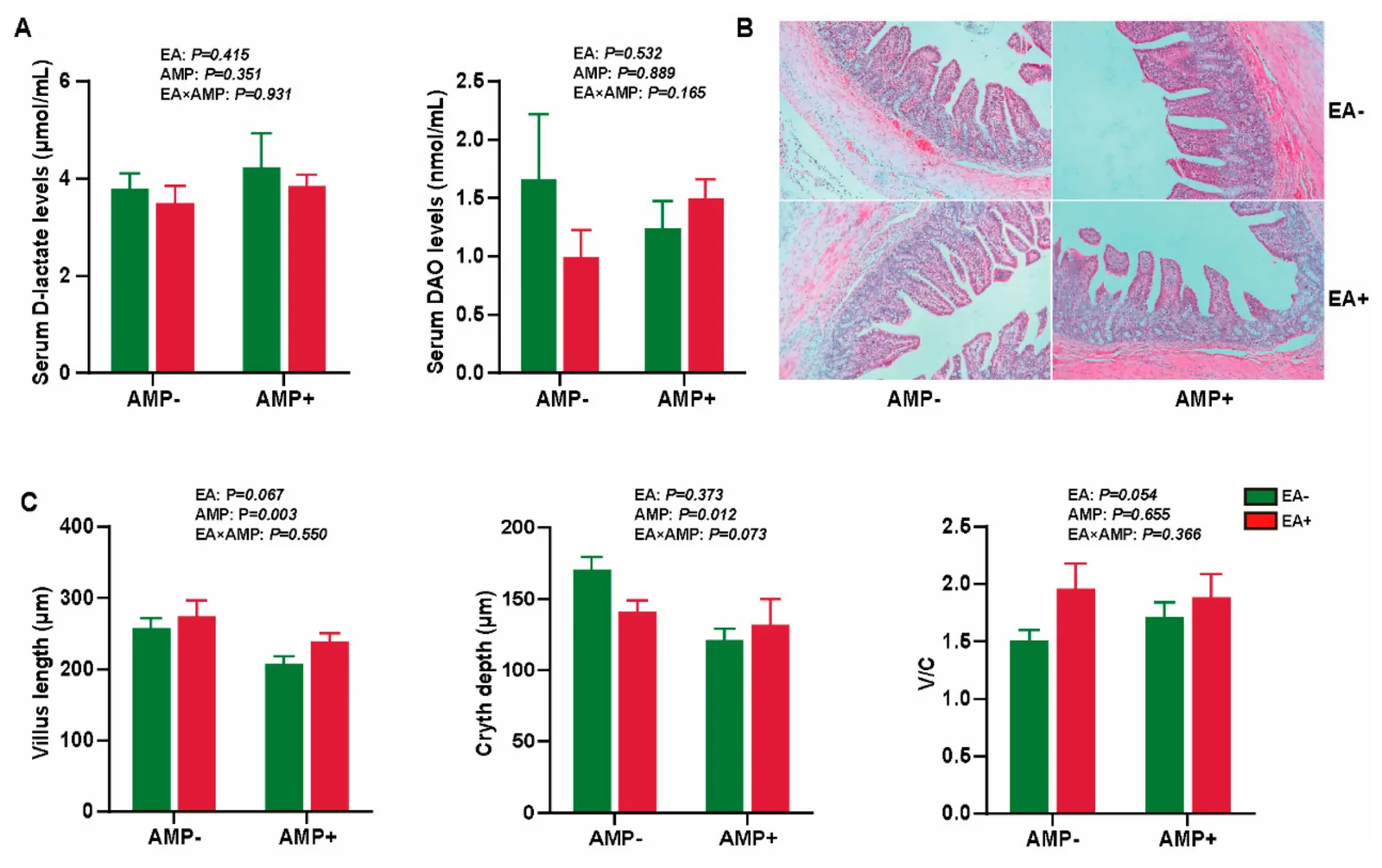
Effects of EA and AMP on serum intestinal-barrier biomarkers and ileal morphological characteristics. (**A**) Serum D-lactate and DAO concentrations; (**B**) Representative H&E-stained images of ileum; (**C**) Ileal morphological parameters including VH, CD, and (VH/CD) ratio. Data are shown as mean ± SEM, *n* = 6 for each group.

The results of intestinal barrier function-related parameters are shown in Figure 3A. Neither EA nor AMP significantly affected serum D-lactate or DAO, and no significant EA × AMP interactions were detected for these markers (*p* > 0.05). The intestinal morphological results are presented in Figure 3B,C. EA supplementation tended to increase the ileal VH/CD ratio (*p* = 0.054) but had no significant main effect on VH or CD (*p* > 0.05). AMP treatment decreased VH and CD (*p* < 0.05), without significantly affecting their ratio (*p* > 0.05).

### 3.6. Effects of EA and AMP on Intestinal Microbial Diversity and Community Composition in PQ-Challenged Piglets

**Figure 4:**
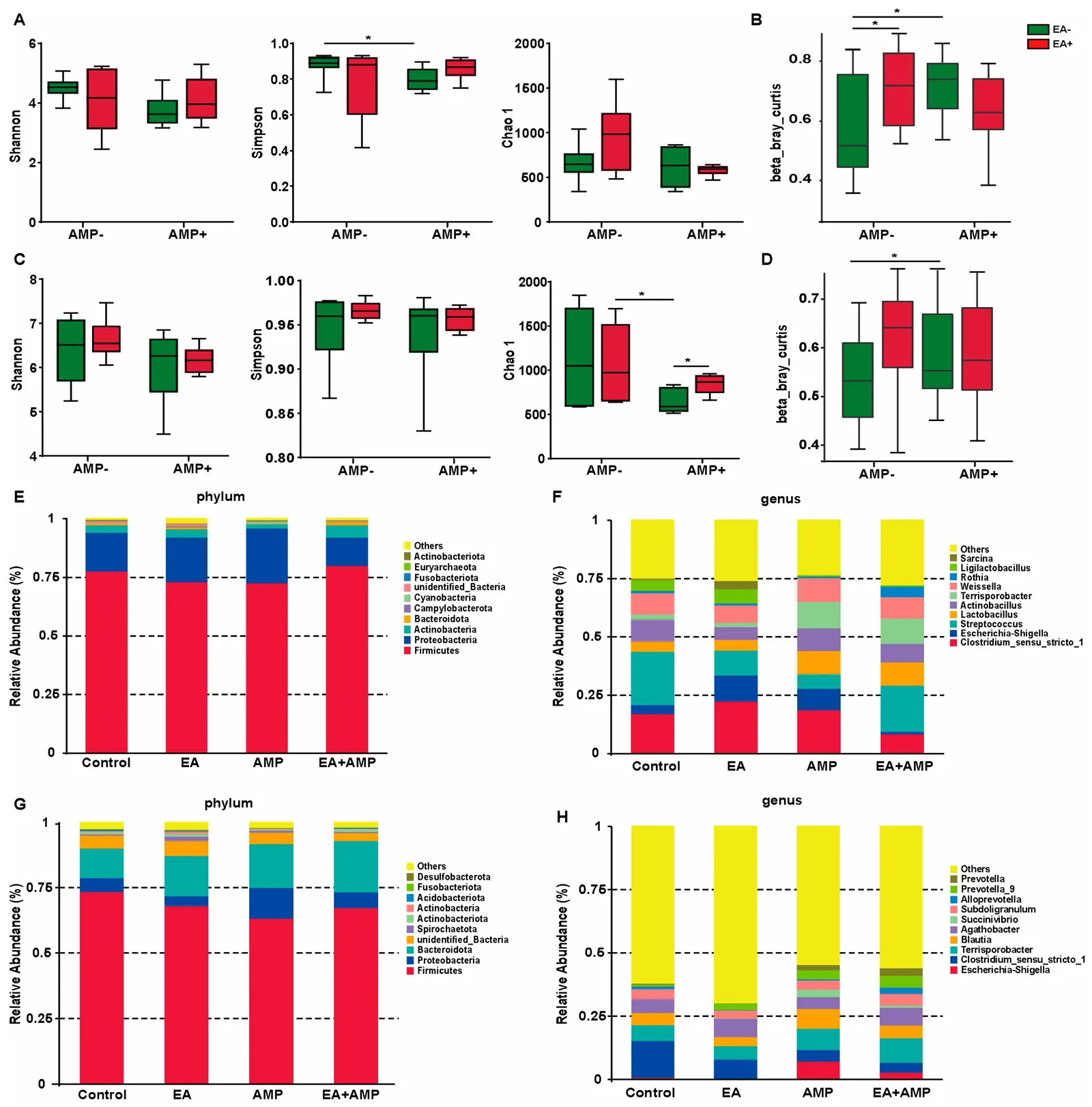
Effects of EA and AMP on gut microbiota diversity and composition in the ileum and colon. (**A**,**B**) Microbial diversity in the ileum. (**C**,**D**) Microbial diversity in the colon. (**E**,**F**) Top 10 abundant microbial taxa at the phylum and genus levels in the ileum. (**G**,**H**) Top 10 abundant microbial taxa at the phylum and genus levels in the colon. Data are shown as mean ± SEM, *n* = 6 for each group. * *p <* 0.05.

Ileal microbial diversity is presented in Figure 4A,B. The Shannon and Chao1 indices did not differ significantly among groups (*p* > 0.05). The Simpson index was lower in the AMP group (AMP+/EA−) than in the Control group (AMP−/EA−; *p* < 0.05; Figure 4A). Bray-Curtis dissimilarity values were higher in the EA group (AMP−/EA+) and the AMP group (AMP+/EA−) than in the Control group (AMP−/EA−; *p* < 0.05; Figure 4B). Firmicutes and Proteobacteria were the predominant ileal phyla. The relative abundance profiles showed lower Firmicutes and higher Proteobacteria in the EA (AMP−/EA+) and AMP (AMP+/EA−) groups than in the Control group (AMP−/EA−), while the AMP + EA group (AMP+/EA+) showed higher Firmicutes and lower Proteobacteria than the AMP group (AMP+/EA−) alone. Predominant genera included *Clostridium_sensu_stricto_1*, *Escherichia-Shigella*, *Streptococcus*, *Lactobacillus*, *Actinobacillus*, and *Terrisporobacter*.

In the colon, Shannon and Simpson indices did not differ significantly among groups (*p* > 0.05; Figure 4C). Chao1 was higher in the AMP + EA group (AMP+/EA+) than in the AMP group (AMP+/EA−; *p* < 0.05). Bray-Curtis dissimilarity values differed between the Control group (AMP−/EA−) and the AMP group (AMP+/EA−; *p* < 0.05; Figure 4D). Firmicutes and Bacteroidota predominated, followed by Proteobacteria and Actinobacteriota (Figure 4G). The relative abundance profiles showed lower Firmicutes and higher Bacteroidota in the EA group (AMP−/EA+) and AMP group (AMP+/EA−) than in the Control group (AMP−/EA−). At the genus level, *Escherichia*-*Shigella* was less abundant in the EA (AMP−/EA+) containing groups, while *Prevotella*, *Prevotella_9*, *Subdoligranulum*, and *Agathobacter* were more abundant in the AMP group (AMP+/EA−) than in the Control group (AMP−/EA−; Figure 4H).

**Figure 5:**
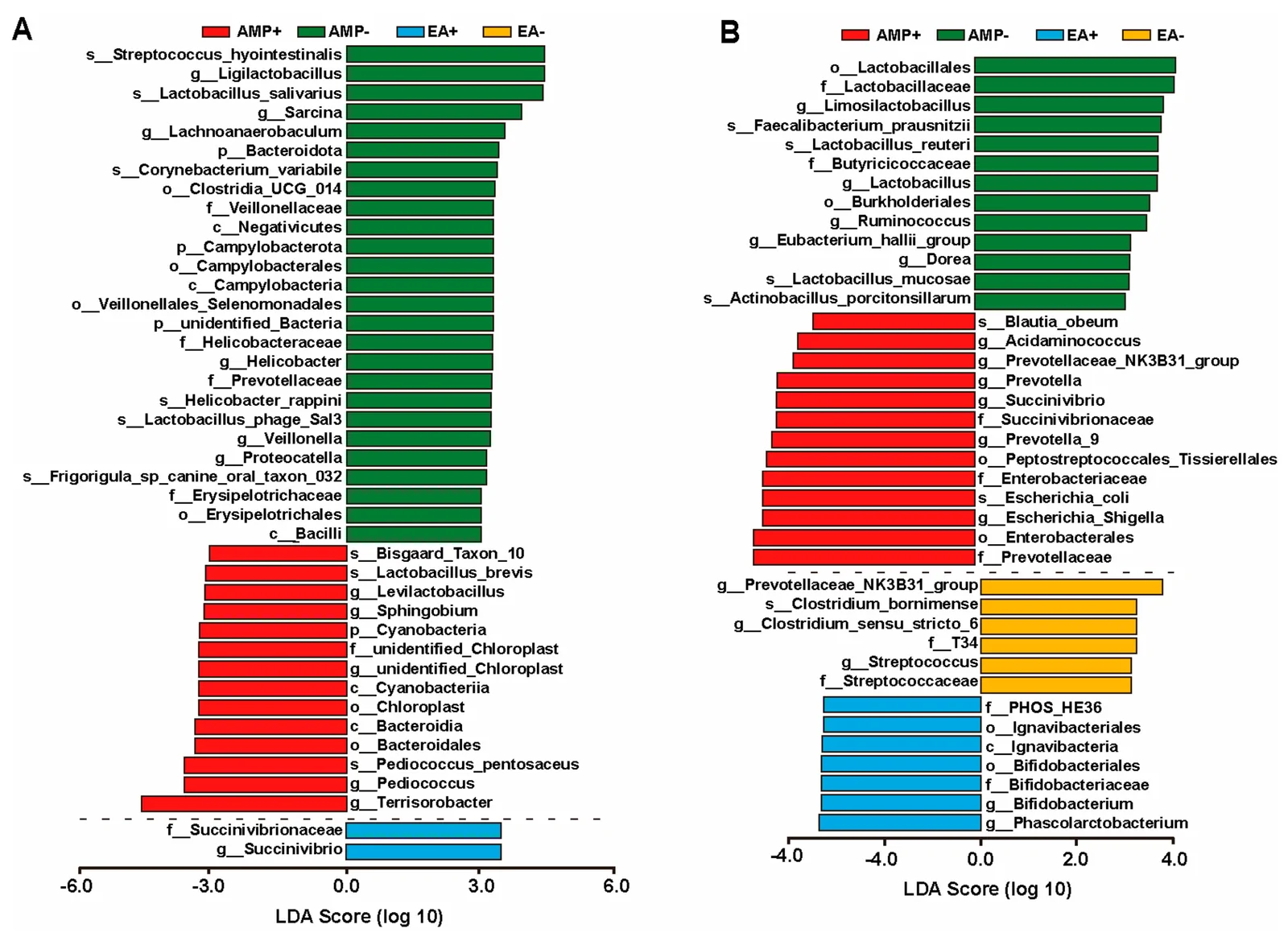
LEfSe analysis of the gut microbiota. (**A**) LEfSe analysis of the ileal microbiota. (**B**) LEfSe analysis of the colonic microbiota. LDA score threshold ≥ 3.0, adjusted *p* < 0.05.

LEfSe analysis identified taxa associated with EA and AMP treatments (Figure 5). In the ileum, *Pediococcus*, *Terrisporobacter*, and *Levilactobacillus* were enriched with AMP treatment, whereas *Ligilactobacillus*, *Helicobacter*, and *Veillonella* were enriched without AMP treatment. *Succinivibrio* was enriched with EA treatment (Figure 5A). In the colon, *Escherichia*-*Shigella*, *Prevotella*, and *Succinivibrio* were enriched with AMP treatment, while *Lactobacillus*, *Limosilactobacillus*, *Ruminococcus*, and *Dorea* were enriched without AMP treatment. *Bifidobacterium* and *Phascolarctobacterium* were enriched with EA treatment, whereas *Streptococcus* and *Clostridium_sensu_stricto_6* were enriched without EA treatment (Figure 5B).

### 3.7. Effects of EA and AMP on Trp Metabolism in PQ-Challenged Piglets

**Figure 6:**
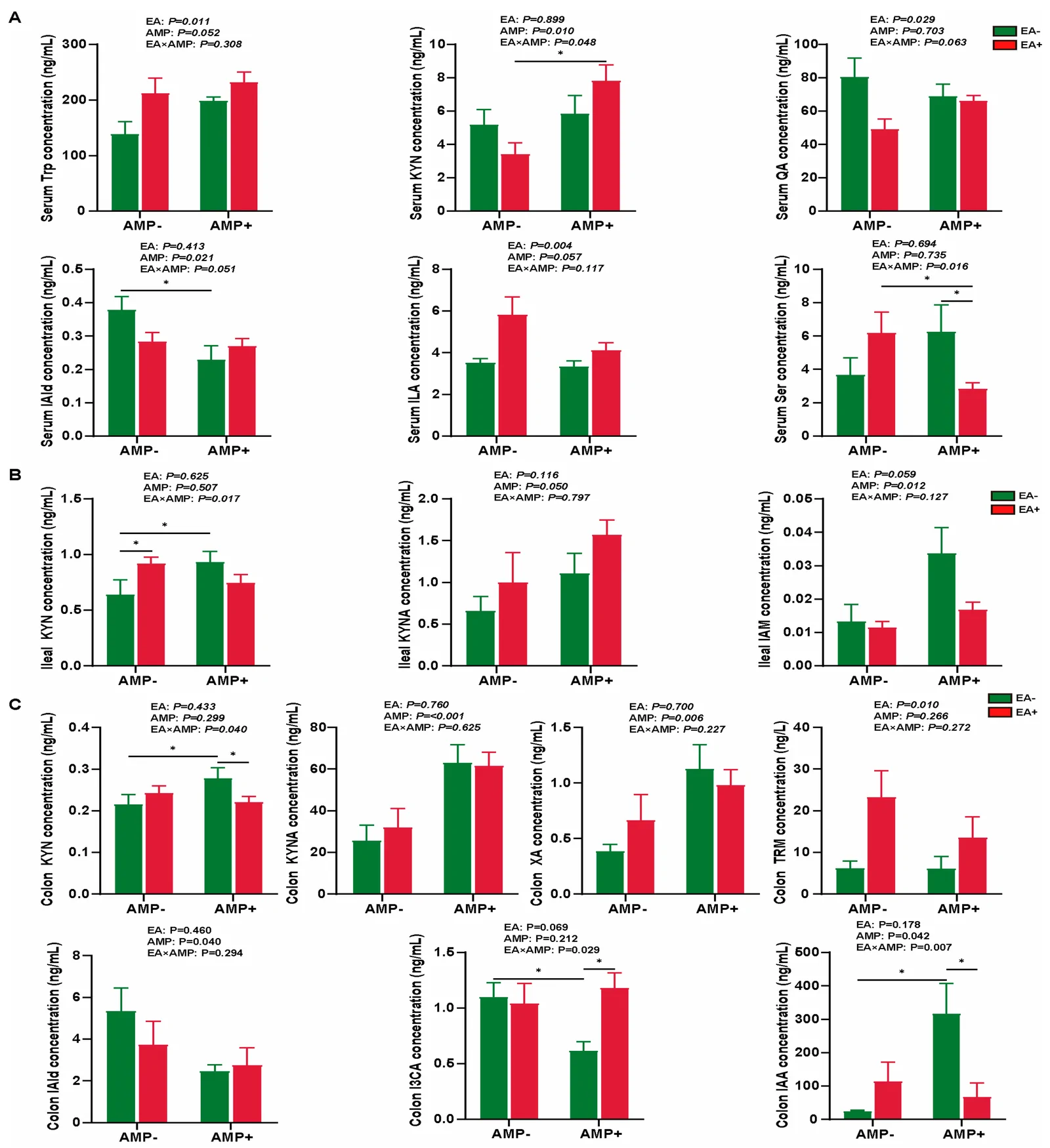
Effects of EA and AMP on Trp metabolism. (**A**) Trp metabolism in serum. (**B**) Trp metabolism in the ileum. (**C**) Trp metabolism in the colon. Data are shown as mean ± SEM, *n* = 6 for each group. * *p* < 0.05.

Serum Trp metabolomic analysis is presented in Table 5 and Figure 6A. EA supplementation increased Trp and ILA and decreased QA (*p* < 0.05). AMP treatment increased KYN, decreased IAld (*p* < 0.05), and tended to increase Trp (*p* = 0.052). Significant EA × AMP interactions were detected for KYN and Ser (*p* < 0.05). Simple effects analysis showed that AMP significantly increased KYN levels and decreased Ser levels in the presence of EA, and EA significantly decreased Ser levels in the presence of AMP (*p* < 0.05).

In the ileum, Trp metabolomic analysis is presented in Table 6 and Figure 6B. EA supplementation had no significant main effect on the measured Trp metabolites (*p* > 0.05). AMP decreased Nam and increased IAM (*p* < 0.05); KYNA concentrations were also higher in AMP-treated groups (*p* = 0.050). A significant EA × AMP interaction was detected for KYN (*p* < 0.05). Simple effects analysis showed that EA significantly increased ileal KYN levels in the absence of AMP, and AMP increased ileal KYN levels in the absence of EA (*p* < 0.05).

In the colon, Trp metabolomic analysis is presented in Table 7 and Figure 6C. EA supplementation increased TRM (*p* < 0.05). AMP increased KYNA, XA, and IAA and decreased IAld (*p* < 0.05). Significant EA × AMP interactions were detected for KYN, I3CA, and IAA (*p* < 0.05). Simple effects analysis showed that AMP significantly increased colonic KYN and IAA levels and decreased I3CA levels in the absence of EA, whereas EA significantly decreased colonic KYN and IAA levels and increased I3CA levels in the presence of AMP (*p* < 0.05).

### 3.8. Correlation Between Gut Microbiota and Trp Metabolites

**Figure 7:**
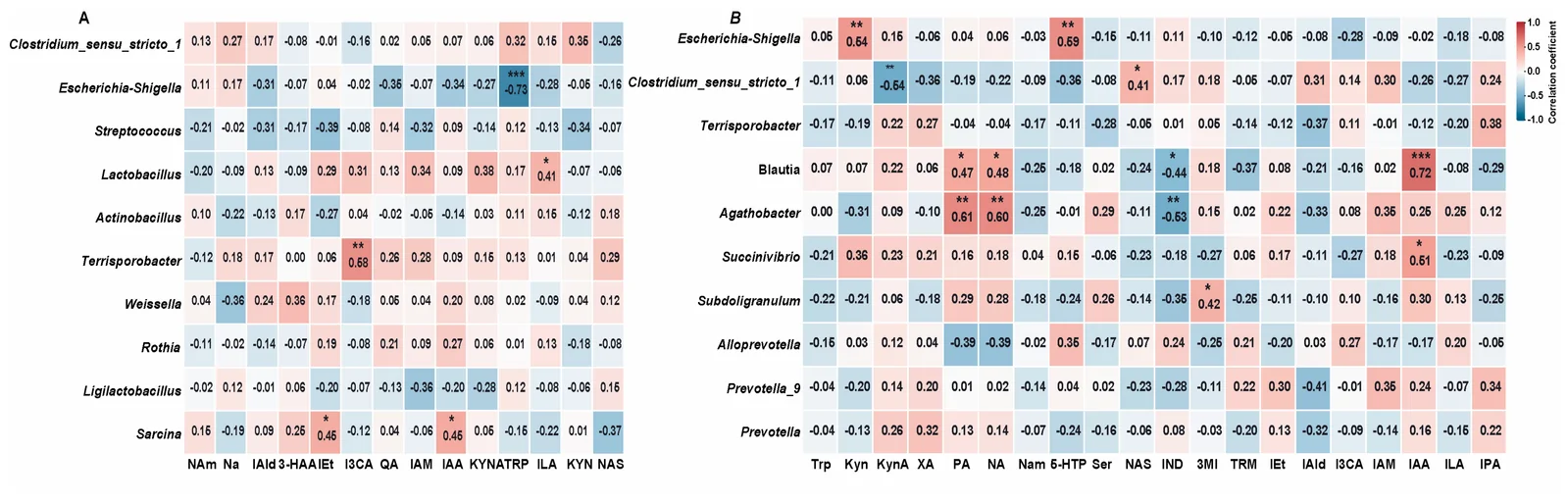
Partial Spearman rank correlations between gut microbial taxa and tryptophan metabolites in the ileum (**A**) and colon (**B**) of 24 piglets, adjusted for treatment group (Control, EA, AMP, and EA + AMP) as a categorical covariate. Red and blue indicate positive and negative correlations, respectively. Asterisks indicate significance based on Benjamini–Hochberg-adjusted *p*-values: * *p* < 0.05, ** *p* < 0.01, and *** *p* < 0.001.

Spearman correlation analysis revealed associations between microbial taxa and Trp metabolites in the ileum and colon (Figure 7). In the ileum, *Escherichia*-*Shigella* was negatively correlated with Trp, whereas *Terrisporobacter* was positively correlated with I3CA. Positive correlations were also observed between *Lactobacillus* and ILA and between *Sarcina* and both IEt and IAA (Figure 7A).

In the colon, *Escherichia*-*Shigella* was positively correlated with Kyn and 5-HTP, whereas *Clostridium_sensu_stricto_1* was negatively correlated with KynA. *Blautia* and *Agathobacter* were positively correlated with PA and NA and negatively correlated with IND. *Blautia* was also positively correlated with IAA. Additional positive correlations were observed between *Clostridium_sensu_stricto_1* and NAS, *Succinivibrio* and IAA, and *Subdoligranulum* and 3MI (Figure 7B).

## 4. Discussion

This study employed a 2 × 2 factorial design to investigate the effects of EA supplementation and AMP treatment on oxidative stress, gut microbiota structure, and Trp metabolism in PQ-challenged piglets. AMP significantly reshaped the microbial communities in the ileum and colon, accompanied by changes in Trp metabolic profiles. The effects of EA on serum MDA and selected metabolites differed according to AMP treatment, indicating that AMP modified certain responses to EA. Concurrent associations between gut microbiota and Trp metabolites suggest that microbiota-associated metabolism may contribute to these responses, although its specific contribution requires further investigation.

The finding most directly related to oxidative stress was a significant EA × AMP interaction for serum MDA: EA supplementation significantly decreased serum MDA in the presence of AMP, whereas no significant effect of EA on MDA was detected in the absence of AMP. MDA is an important end product of lipid peroxidation and is commonly used as a marker of lipid oxidative damage. PQ can induce oxidative damage by promoting reactive oxygen species generation and initiating lipid peroxidation chain reactions [28,29,30]. The reduction in serum MDA with EA supplementation in the presence of AMP therefore suggests lower lipid peroxidation under these experimental conditions. However, T-AOC did not differ significantly among groups, and the effects of EA on growth performance and serum markers related to intestinal barrier integrity, including D-lactate and DAO, did not reach statistical significance. Thus, the observed antioxidant response to EA was primarily reflected in MDA, providing insufficient evidence to infer broad improvements in overall antioxidant capacity or intestinal barrier function.

Previous studies have shown that EA possesses free radical-scavenging activity and can contribute to antioxidant defenses through the regulation of signaling pathways such as Nrf2/ARE and NF-κB [9,12,13]. In addition, EA is poorly absorbed in the upper gastrointestinal tract, and a proportion reaches the hindgut, where intestinal microorganisms can convert it into biologically active metabolites, including urolithins [31,32]. These bioavailability and metabolic characteristics provide a possible explanation for the different responses to EA in the presence and absence of AMP. AMP-associated microbial changes may influence EA biotransformation and the resulting metabolite profile. However, because EA and urolithins were not directly measured, this explanation remains a hypothesis requiring further validation.

The gut microbiota is an important target of dietary components and antibiotics. In this study, AMP significantly altered microbial diversity and community structure in the ileum and colon. Specifically, the ileal Simpson index decreased significantly, and microbial composition exhibited distinct changes across intestinal segments, indicating that AMP induced segment-specific microbial perturbations under the present experimental conditions. LEfSe analysis further supported these segment-specific changes, identifying enrichment of *Terrisporobacter* and *Pediococcus* in the ileum and Enterobacteriaceae and *Escherichia*-*Shigella* in the colon of AMP+ animals. These findings are consistent with previous reports that antibiotics can suppress susceptible microbial populations and alter ecological competition, thereby increasing the relative abundance of certain taxa [33,34,35,36].

The different microbial responses across intestinal segments may be related to differences in physiological conditions, substrate concentrations, oxygen partial pressures, and immune status. Previous studies have described bidirectional interactions between EA and the gut microbiota: intestinal microorganisms can convert EA into metabolites such as urolithins, while EA and its metabolites can influence microbial composition [13,31,32]. In the present study, in the absence of AMP, EA increased the abundance of ileal lactobacilli, including Lactobacillus salivarius. In the presence of AMP, EA supplementation shifted the relative proportions of Firmicutes and Proteobacteria in the opposite direction to the AMP-associated changes, accompanied by enrichment of Prevotella taxa. These findings indicate that EA can influence selected microbial taxa under different AMP treatment conditions, although whether these compositional changes are accompanied by functional improvements remains to be determined. Intestinal morphological analysis showed that AMP significantly reduced ileal villus height and crypt depth, whereas serum markers related to intestinal barrier damage did not increase significantly. This suggests that intestinal morphological changes were not fully reflected in the measured serum markers; the absence of significant changes in these markers therefore does not exclude local barrier impairment. The effects of EA on intestinal morphology did not reach statistical significance, and their functional relationship with microbial changes remains unclear.

Trp is an important link between host metabolism and gut microbial function and is primarily metabolized through the kynurenine, serotonin, and indole pathways. The kynurenine and serotonin pathways predominantly involve host metabolism, whereas intestinal microorganisms contribute to the production of various indole derivatives. These microbiota-associated metabolites play important roles in intestinal immunity and homeostasis [18,37,38]. In this study, AMP significantly altered Trp metabolic profiles in serum and different intestinal segments, with distinct patterns across sampling sites. In serum, AMP mainly affected KYN and IAld levels. In the intestine, AMP primarily affected KYNA, IAM, and Nam in the ileum and KYNA, XA, and IAA in the colon. The effects of EA on selected Trp metabolites also differed according to AMP treatment. EA decreased serum KYN in the absence of AMP but increased it in the presence of AMP. Interactions were also observed for Ser, Cit, and Cys. These findings indicate that AMP modified the direction or magnitude of the effects of EA on selected metabolic outcomes. Because AMP also altered microbial composition, microbial metabolism is one possible explanation for these differences, although potential contributions from host metabolism and other effects of AMP should also be considered. Differences in metabolic responses across intestinal segments may be related to microbial metabolic capacity, substrate availability, the local metabolic environment, and host enzyme expression [18,37]. Nevertheless, metabolite concentrations reflect the dynamic balance among production, utilization, absorption, and clearance; changes in metabolite concentrations alone therefore cannot directly establish changes in the activity of specific metabolic pathways.

Correlation analysis revealed segment-specific associations between gut microbial taxa and Trp metabolites. In the ileum, *Lactobacillus* and *Terrisporobacter* were positively correlated with ILA and I3CA, respectively, whereas *Escherichia*-*Shigella* was negatively correlated with Trp. In the colon, *Blautia* and *Succinivibrio* were positively correlated with IAA, while *Blautia* and *Agathobacter* showed positive correlations with PA and NA and negative correlations with IND. These patterns highlight distinct taxon–metabolite relationships across intestinal segments and provide candidates for further functional investigation. Previous studies have linked microbial Trp-derived indole metabolites, including IAA, IAld, and ILA, to AhR signaling and the regulation of intestinal immunity and barrier function [37,38,39]. Together with the microbial and metabolic changes observed here, these findings suggest that microbiota-associated Trp metabolism may participate in responses to EA and AMP. However, even after adjustment for treatment, these associations cannot identify metabolite-producing strains, distinguish microbial production from consumption, or establish that the microbiota mediates EA’s effects. Moreover, AhR signaling was not examined in this study. The proposed microbial and signaling mechanisms therefore remain hypotheses requiring functional validation.

Several limitations should be acknowledged. First, all piglets received PQ, and no non-PQ control was included. Thus, the findings are restricted to the PQ-challenged model and cannot establish the magnitude of PQ-induced changes or whether EA reversed or AMP exacerbated these changes. Second, the small sample size may limit statistical power, particularly for interactions; nonsignificant results should not be interpreted as evidence of no effect. Third, replicate measurements were not performed to assess intra- and inter-assay precision, limiting the evaluation of analytical variability and measurement reproducibility. Finally, EA and urolithins were not measured, and microbial functions and AhR, Nrf2, and NF-κB signaling were not examined. Their roles in the observed responses, as well as the necessity of the microbiota for EA’s antioxidant effect, therefore remain unconfirmed and require functional validation.

## 5. Conclusions

In PQ-challenged piglets, EA supplementation significantly decreased serum MDA in the presence of AMP, while AMP altered gut microbial composition and Trp metabolic profiles. The effects of EA on selected metabolites differed according to AMP treatment. These findings indicate that AMP modified certain responses to EA, which coincided with microbial and metabolic changes. The potential contribution of microbiota-associated Trp metabolism to these responses requires further functional validation.

## Figures and Tables

**Table 1:** Ingredient composition of diets.

Items	Percent (%)
Corn	32.20
Extruded corn	30.00
Soybean meal	10.14
Fermented soybean	8.00
Whey powder	5.00
Fish meal	5.00
Soybean oil	2.00
Glucose	3.00
L-lysine	0.55
DL-methionine	0.07
L-threonine	0.20
L-tryptophan	0.04
Limestone	0.20
Calcium hydrophosphate	0.40
Calcium Formate	0.60
Sodium chloride	0.40
Citric acid	0.80
Choline chloride	0.10
ZnO	0.20
Antioxidant	0.10
Premix ^1^	1.00
Total	100.00

^1^ The premix provided the following per kg of diets: Zn (ZnO) 50.00 mg, Cu (CuSO_4_) 20.00 mg, Mn (MnO) 55.00 mg, Fe (FeSO_4_) 100.00 mg, I (KI) 1.00 mg, Co (CoSO_4_) 2.00 mg, Se (Na_2_SeO_3_) 0.30 mg, VA 8255.00 IU, VD3 2000.00 IU, VE 40.00 IU, VB1 2.00 mg, VB2 4.00 mg, VC 100.00 mg, VK3 1.50 mg, VB6 10.00 mg, VB12 0.05 mg, nicotinic acid 30.00 mg, folic acid 2.00 mg, biotin 0.20 mg, and pantothenic acid 15.00 mg.

**Table 2:** Growth performance of piglets.

Item	EA		AMP		SEM	*p*-Value		
	−	+	−	+		EA	AMP	EA × AMP
Body weight (kg)								
Day 0	7.54	7.30	7.34	7.50	0.280	0.562	0.678	0.934
Day 14	10.10	9.80	9.49	10.42	0.518	0.687	0.218	0.858
Day 21	11.78	11.62	11.13	12.27	0.677	0.863	0.250	0.706
ADG (g/d)	202.38	205.56	180.95	226.99	29.398	0.940	0.281	0.652
ADFI (g/d)	662.70	641.27	635.71	668.25	35.621	0.675	0.526	0.264
Organ indexes								
Heart (g/kg)	8.04	8.26	7.72	8.58	0.506	0.756	0.239	0.638
Liver (g/kg)	22.51	24.74	23.22	24.03	0.769	0.053	0.468	0.141
Spleen (g/kg)	2.25	2.38	2.19	2.44	0.121	0.473	0.153	0.309
Kidney (g/kg)	5.96	6.44	5.97	6.43	0.306	0.287	0.308	0.092

**Table 3:** Effects of EA and AMP and their interaction on serum biochemical parameters in PQ-challenged piglets.

Item	EA		AMP		SEM	*p*-Value		
	−	+	−	+		EA	AMP	EA × AMP
ALT (U/L)	53.34	54.81	54.79	53.36	3.047	0.720	0.744	0.737
AST (U/L)	85.52	79.11	79.94	84.69	5.959	0.056	0.579	0.456
ALP (U/L)	192.84	198.93	196.20	195.57	14.583	0.557	0.976	0.771
TP (g/L)	56.74	62.29	56.61	62.41	1.990	0.500	0.053	0.063
ALB (g/L)	30.53	34.65	30.12	35.06	1.428	0.857	0.024	0.055
GLB (g/L)	26.17	27.50	26.34	27.33	1.678	0.491	0.678	0.581
GLU (mmol/L)	5.08	4.80	5.34	4.54	0.363	0.599	0.138	0.578
BUN (mmol/L)	2.68	2.94	2.92	2.70	0.270	0.192	0.576	0.492
TC (mmol/L)	2.30	2.46	2.43	2.33	0.106	0.353	0.530	0.268
TG (mmol/L)	0.35	0.41	0.44	0.32	0.024	0.058	0.001	0.077
HDL-C (mmol/L)	0.81	0.89	0.88	0.83	0.057	0.290	0.553	0.321
LDL-C (mmol/L)	1.08	1.15	1.18	1.05	0.074	0.613	0.249	0.469

**Table 4:** Effects of EA and AMP and their interaction on serum amino acid profiles in PQ-challenged piglets.

Item	EA		AMP		SEM	*p*-Value		
	−	+	−	+		EA	AMP	EA × AMP
Ala	21.55	26.99	24.58	23.96	1.370	0.011	0.753	0.482
Cit	5.30	4.82	5.21	4.91	0.298	0.277	0.490	0.025
Val	7.50	7.45	7.46	7.50	0.820	0.966	0.972	0.329
Cys	1.01	1.19	0.84	1.36	0.183	0.515	0.056	0.030
Met	4.25	5.08	4.46	4.87	0.290	0.057	0.327	0.376
Ile	4.80	4.87	4.81	4.86	0.432	0.910	0.941	0.961
Leu	10.64	11.31	10.64	11.31	0.742	0.531	0.528	0.433
Tyr	6.73	7.29	6.73	7.28	0.686	0.571	0.575	0.639
Phe	7.16	6.40	6.79	6.77	0.342	0.132	0.968	0.983
Orn	6.10	6.42	6.08	6.44	0.395	0.573	0.529	0.253
Lys	26.99	30.39	28.85	28.53	2.201	0.288	0.919	0.404
His	4.48	4.23	4.92	3.79	0.525	0.743	0.144	0.113
Car	0.75	0.61	0.80	0.56	0.082	0.226	0.052	0.657
Arg	9.37	9.74	10.09	9.02	1.043	0.807	0.476	0.207
Pro	12.62	14.82	13.98	13.46	0.718	0.042	0.611	0.669
Tau	6.10	6.11	6.00	6.21	0.593	0.995	0.805	0.897
Asp	1.23	1.51	1.57	1.17	0.170	0.254	0.118	0.954
Thr	15.44	16.20	16.14	15.50	1.566	0.734	0.776	0.998
Ser	6.55	7.89	6.99	7.45	0.631	0.152	0.609	0.471
Glu	15.24	17.84	16.84	16.23	1.234	0.152	0.731	0.744
Gly	39.16	50.66	44.12	45.70	4.779	0.104	0.818	0.884

**Table 5:** Effects of EA and AMP and their interaction on serum Trp metabolism in PQ-challenged piglets.

Item	EA		AMP		SEM	*p*-Value		
	−	+	−	+		EA	AMP	EA × AMP
Trp	169.37	223.08	176.45	216.00	28.436	0.011	0.052	0.308
KYN	5.54	5.66	4.34	6.86	1.281	0.899	0.010	0.048
KYNA	0.18	0.23	0.21	0.20	0.023	0.152	0.631	0.344
HK	0.24	0.28	0.25	0.27	0.021	0.447	0.673	0.697
QA	75.05	58.01	65.13	67.94	9.160	0.029	0.703	0.063
Nam	11.69	13.60	11.39	13.90	1.846	0.459	0.331	0.214
IAld	0.31	0.28	0.33	0.25	0.045	0.413	0.021	0.051
IAA	8.79	10.85	9.85	9.79	0.856	0.430	0.982	0.885
IPA	2.07	2.33	2.56	1.83	0.491	0.655	0.220	0.125
ILA	3.45	5.00	4.70	3.75	0.805	0.004	0.057	0.117
5-HTP	0.16	0.16	0.16	0.16	0.016	0.643	0.803	0.095
Ser	4.99	4.54	4.96	4.57	1.236	0.694	0.735	0.016

*p*-values were adjusted using the Benjamini–Hochberg procedure, with all metabolite-related tests within each table treated as a separate family of comparisons.

**Table 6:** Effects of EA and AMP and their interaction on ileal Trp metabolism in PQ-challenged piglets.

Item	EA		AMP		SEM	*p*-Value		
	−	+	−	+		EA	AMP	EA × AMP
Nam	0.90	0.78	1.27	0.41	0.272	0.751	0.038	0.391
Na	9.44	12.19	10.69	10.93	1.298	0.150	0.898	0.709
IAld	0.41	0.43	0.40	0.44	0.071	0.855	0.718	0.249
3-HAA	0.07	0.08	0.07	0.08	0.023	0.751	0.724	0.051
IEt	0.17	0.25	0.20	0.22	0.048	0.228	0.660	0.357
I3CA	0.20	0.19	0.13	0.27	0.054	0.891	0.082	0.711
QA	2.97	4.23	2.94	4.27	0.609	0.159	0.139	0.864
IAM	0.020	0.015	0.010	0.025	0.0035	0.059	0.012	0.127
IAA	14.40	18.10	15.16	17.34	2.954	0.387	0.608	0.492
KYNA	0.89	1.30	0.84	1.35	0.173	0.116	0.050	0.797
Trp	233.85	235.72	225.32	244.25	11.613	0.910	0.263	0.487
ILA	0.95	1.48	1.02	1.41	0.305	0.231	0.372	0.943
KYN	0.79	0.84	0.78	0.85	0.063	0.625	0.507	0.017
NAS	0.020	0.020	0.020	0.020	0.0007	0.624	0.394	1.000

*p*-values were adjusted using the Benjamini–Hochberg procedure, with all metabolite-related tests within each table treated as a separate family of comparisons.

**Table 7:** Effects of EA and AMP and their interaction on colon Trp metabolism in PQ-challenged piglets.

Item	EA		AMP		SEM	*p*-Value		
	−	+	−	+		EA	AMP	EA × AMP
Trp	138.11	126.76	145.78	119.08	12.724	0.535	0.153	0.086
KYN	0.25	0.23	0.23	0.25	0.013	0.433	0.299	0.040
KYNA	44.64	47.01	29.08	62.57	5.462	0.762	<0.001	0.625
XA	0.76	0.83	0.53	1.06	0.121	0.700	0.006	0.227
PA	729.42	738.25	723.34	744.33	43.364	0.887	0.736	0.989
NA	289.84	291.67	286.25	295.25	17.611	0.942	0.722	0.853
Nam	0.14	0.18	0.17	0.15	0.026	0.264	0.510	0.912
5-HTP	0.305	0.320	0.295	0.330	0.037	0.706	0.537	0.798
Ser	1.28	1.44	1.45	1.26	0.126	0.379	0.299	0.067
NAS	0.050	0.040	0.055	0.035	0.009	0.717	0.204	0.212
IND	40.76	39.44	40.83	39.36	5.962	0.877	0.863	0.223
3MI	44.80	41.81	48.45	38.15	7.609	0.784	0.350	0.401
TRM	6.32	18.51	14.86	9.98	3.017	0.010	0.266	0.272
IEt	1.60	1.92	1.80	1.72	0.182	0.222	0.756	0.057
IAld	3.93	3.27	4.57	2.63	0.622	0.460	0.040	0.294
I3CA	0.86	1.12	1.07	0.91	0.093	0.069	0.212	0.029
IAM	0.07	0.06	0.07	0.06	0.009	0.585	0.432	0.821
IAA	171.58	92.51	70.55	193.53	40.029	0.178	0.042	0.007
ILA	1.49	2.23	2.40	1.32	0.372	0.180	0.053	0.553
IPA	3.48	4.51	3.87	4.12	0.613	0.247	0.785	0.434

*p*-values were adjusted using the Benjamini–Hochberg procedure, with all metabolite-related tests within each table treated as a separate family of comparisons.

## Data Availability

Data Availability Statement: The datasets presented in this study can be found in online repositories. The names of the repository/repositories and accession number(s) can be found at: https://www.ncbi.nlm.nih.gov/bioproject/PRJNA1527587 (accessed on 3 September 2026).

## Supplementary Materials

The following supporting information can be downloaded at: https://www.mdpi.com/article/10.3390/antiox15101356/s1, File S1: Targeted quantification of tryptophan and its metabolites; Table S1: Tryptophan-related standards used in the assay; Table S2: Instruments used for metabolite extraction; Table S3: LC-MS/MS system; Table S4: Standard curve concentration of tryptophan; Table S5: MRM transitions and mass spectrometric parameters for the quantified analytes; Table S6: Intra-batch, inter-batch and total precision, repeatability, and stability; Table S7: Matrix effects and recovery; Table S8: QC stability in colonic and ileum digesta and serum; Table S9: Comprehensive data of tryptophan quantification, microbial diversity, relative abundance, and correlation analysis.

## Abbreviations

OS	Oxidative Stress
ROS	Reactive Oxygen Species
LPS	Lipopolysaccharide
EA	Ellagic acid
Trp	Tryptophan
PQ	Paraquat
AMP	Ampicillin
MDA	Malondialdehyde

## References

[1] Sies H., Berndt C., Jones D.P. (2017). Oxidative Stress. Annu. Rev. Biochem..

[2] Schieber M., Chandel N.S. (2014). ROS function in redox signaling and oxidative stress. Curr. Biol..

[3] Sun Y., Wang X., Li L., Zhong C., Zhang Y., Yang X., Li M., Yang C. (2024). The role of gut microbiota in intestinal disease: From an oxidative stress perspective. Front. Microbiol..

[4] Li L., Peng P., Ding N., Jia W., Huang C., Tang Y. (2023). Oxidative Stress, Inflammation, Gut Dysbiosis: What Can Polyphenols Do in Inflammatory Bowel Disease?. Antioxidants.

[5] Shyanti R.K., Greggs J., Malik S., Mishra M. (2024). Gut dysbiosis impacts the immune system and promotes prostate cancer. Immunol. Lett..

[6] Durack J., Lynch S.V. (2018). The gut microbiome: Relationships with disease and opportunities for therapy. J. Exp. Med..

[7] Cani P.D., Amar J., Iglesias M.A., Poggi M., Knauf C., Bastelica D., Neyrinck A.M., Fava F., Tuohy K.M., Chabo C. (2007). Metabolic endotoxemia initiates obesity and insulin resistance. Diabetes.

[8] Ríos J.-L., Giner R.M., Marín M., Recio M.C. (2018). A Pharmacological Update of Ellagic Acid. Planta Med..

[9] Xiao Y., Huang R., Wang N., Deng Y., Tan B., Yin Y., Qi M., Wang J. (2022). Ellagic Acid Alleviates Oxidative Stress by Mediating Nrf2 Signaling Pathways and Protects against Paraquat-Induced Intestinal Injury in Piglets. Antioxidants.

[10] Espín J.C., González-Sarrías A., Tomás-Barberán F.A. (2017). The gut microbiota: A key factor in the therapeutic effects of (poly)phenols. Biochem. Pharmacol..

[11] Selma M.V., Espín J.C., Tomás-Barberán F.A. (2009). Interaction between phenolics and gut microbiota: Role in human health. J. Agric. Food Chem..

[12] Ribeiro M., Alvarenga L., Cardozo L.F.M.F., Baptista B.G., Nascimento D., Esgalhado M., Mafra D. (2025). Urolithin as a Metabolite of Ellagitannins and Ellagic Acid from Fruits and Nuts Produced by the Gut Microbiota: Its Role on Non-Communicable Diseases. Curr. Nutr. Rep..

[13] Leng P., Wang Y., Xie M. (2025). Ellagic Acid and Gut Microbiota: Interactions, and Implications for Health. Food Sci. Nutr..

[14] Tomás-Barberán F.A., Selma M.V., Espín J.C. (2016). Interactions of gut microbiota with dietary polyphenols and consequences to human health. Curr. Opin. Clin. Nutr. Metab. Care.

[15] Roager H.M., Licht T.R. (2018). Microbial tryptophan catabolites in health and disease. Nat. Commun..

[16] Mester P., Steinmann S.M., Mehler S., Müller M., Gülow K. (2026). Microbiota-Derived Metabolites in the Epigenetic Regulation of Redox Homeostasis. Antioxidants.

[17] Agus A., Clément K., Sokol H. (2020). Gut microbiota-derived metabolites as central regulators in metabolic disorders. Gut.

[18] Gao J., Xu K., Liu H., Liu G., Bai M., Peng C., Li T., Yin Y. (2018). Impact of the Gut Microbiota on Intestinal Immunity Mediated by Tryptophan Metabolism. Front. Cell. Infect. Microbiol..

[19] Zelante T., Iannitti R.G., Cunha C., De Luca A., Giovannini G., Pieraccini G., Zecchi R., D’Angelo C., Massi-Benedetti C., Fallarino F. (2013). Tryptophan catabolites from microbiota engage aryl hydrocarbon receptor and balance mucosal reactivity via interleukin-22. Immunity.

[20] Hubbard T.D., Murray I.A., Perdew G.H. (2015). Indole and Tryptophan Metabolism: Endogenous and Dietary Routes to Ah Receptor Activation. Drug Metab. Dispos..

[21] Rothhammer V., Quintana F.J. (2019). The aryl hydrocarbon receptor: An environmental sensor integrating immune responses in health and disease. Nat. Rev. Immunol..

[22] Stockinger B., Di Meglio P., Gialitakis M., Duarte J.H. (2014). The aryl hydrocarbon receptor: Multitasking in the immune system. Annu. Rev. Immunol..

[23] Liu Z., Zhao F., Li Q., Shang Q., Fang D., Li X., Li H., He Q., Zhang D., Cheng J. (2026). Multi-omics chemical and biochemical profiling reveals ellagic acid enhances insulin sensitivity via gut microbiota-tryptophan-indole signaling mechanism. Food Chem..

[24] Kim Y.-S., Zerin T., Song H.-Y. (2013). Antioxidant action of ellagic acid ameliorates paraquat-induced A549 cytotoxicity. Biol. Pharm. Bull..

[25] Zhang X., Wang S., Wu Y., Liu X., Wang J., Han D. (2022). Ellagic Acid Alleviates Diquat-Induced Jejunum Oxidative Stress in C57BL/6 Mice through Activating Nrf2 Mediated Signaling Pathway. Nutrients.

[26] Fussell K.C., Udasin R.G., Gray J.P., Mishin V., Smith P.J.S., Heck D.E., Laskin J.D. (2011). Redox cycling and increased oxygen utilization contribute to diquat-induced oxidative stress and cytotoxicity in Chinese hamster ovary cells overexpressing NADPH-cytochrome P450 reductase. Free Radic. Biol. Med..

[27] Shi Y., Kellingray L., Zhai Q., Gall G.L., Narbad A., Zhao J., Zhang H., Chen W. (2018). Structural and Functional Alterations in the Microbial Community and Immunological Consequences in a Mouse Model of Antibiotic-Induced Dysbiosis. Front. Microbiol..

[28] Suntres Z.E. (2002). Role of antioxidants in paraquat toxicity. Toxicology.

[29] Blanco-Ayala T., Andérica-Romero A.C., Pedraza-Chaverri J. (2014). New insights into antioxidant strategies against paraquat toxicity. Free Radic. Res..

[30] Tsikas D. (2016). Assessment of lipid peroxidation by measuring malondialdehyde (MDA) and relatives in biological samples: Analytical and biological challenges. Anal. Biochem..

[31] Espín J.C., Larrosa M., García-Conesa M.T., Tomás-Barberán F. (2013). Biological significance of urolithins, the gut microbial ellagic Acid-derived metabolites: The evidence so far. Evid. Based Complement. Altern. Med..

[32] García-Villalba R., Giménez-Bastida J.A., Cortés-Martín A., Ávila-Gálvez M.Á., Tomás-Barberán F.A., Selma M.V., Espín J.C., González-Sarrías A. (2022). Urolithins: A Comprehensive Update on their Metabolism, Bioactivity, and Associated Gut Microbiota. Mol. Nutr. Food Res..

[33] Maier L., Goemans C.V., Wirbel J., Kuhn M., Eberl C., Pruteanu M., Müller P., Garcia-Santamarina S., Cacace E., Zhang B. (2021). Unravelling the collateral damage of antibiotics on gut bacteria. Nature.

[34] Kers J.G., Saccenti E. (2022). The Power of Microbiome Studies: Some Considerations on Which Alpha and Beta Metrics to Use and How to Report Results. Front. Microbiol..

[35] Santacroce L., Di Domenico M., Montagnani M., Jirillo E. (2023). Antibiotic Resistance and Microbiota Response. Curr. Pharm. Des..

[36] Pennycook J.H., Scanlan P.D. (2021). Ecological and Evolutionary responses to Antibiotic Treatment in the Human Gut Microbiota. FEMS Microbiol. Rev..

[37] Agus A., Planchais J., Sokol H. (2018). Gut Microbiota Regulation of Tryptophan Metabolism in Health and Disease. Cell Host Microbe.

[38] Scott S.A., Fu J., Chang P.V. (2020). Microbial tryptophan metabolites regulate gut barrier function via the aryl hydrocarbon receptor. Proc. Natl. Acad. Sci. USA.

[39] Gheorghe C.E., Martin J.A., Manriquez F.V., Dinan T.G., Cryan J.F., Clarke G. (2019). Focus on the essentials: Tryptophan metabolism and the microbiome-gut-brain axis. Curr. Opin. Pharmacol..

