# Supplementary material for: Effects of Ellagic Acid on Gut Microbiota and Tryptophan Metabolism in Paraquat-Challenged Piglets Subjected to Ampicillin-Induced Microbiota Perturbation
Source: Antioxidants (Basel). 2026 Oct 20;15(10):1356. doi: 10.3390/antiox15101356 (PMC13701896; doi:10.3390/antiox15101356)
Supplement: Supplementary file 1 [file antioxidants-15-01356-s001.zip › File S1-Targeted quantification of tryptophan and its metabolites.pdf]

Materials and Methods

1.1 Reagents and materials

Methanol (HPLC grade, ≥ 99.0%) was purchased from Thermo Fisher Scientific; formic acid (LC-MS grade) was obtained from TCI; and ultrapure water was purified by a Millipore system. All 30 tryptophan-related standards and the isotopically labeled internal standard (tryptophan-d3, Trp-d3) are listed in Table 1. The instruments used for metabolite extraction and instrumental analysis are listed in Tables 2 and 3, respectively.

Table S1. Tryptophan-related standards used in the assay

Abbrev.	Compound	CAS No.
2-AP	2-Aminophenol	95-55-6
IND	Indole	120-72-9
NAm	Nicotinamide	98-92-0
NA	Nicotinic acid	59-67-6
PA	Picolinic acid	98-98-6
3MI	3-Methylindole (skatole)	83-34-1
IAld	Indole-3-carboxaldehyde	487-89-8
5-HAA	5-Hydroxyanthranilic acid	394-31-0
3-HAA	3-Hydroxyanthranilic acid	548-93-6
TRM	Tryptamine	61-54-1
IEt	Tryptophol	526-55-6
I3CA	Indole-3-carboxylic acid	771-50-6
QA	Quinolinic acid	89-00-9
IAM	Indole-3-acetamide	879-37-8
IAA	Indole-3-acetic acid	87-51-4
SER	Serotonin	50-67-9
IArA	Indole-3-acrylic acid	1204-06-4
IPA	Indole-3-propionic acid	830-96-6
KYNA	Kynurenic acid	492-27-3
MeOTA	5-Methoxytryptamine	608-07-1
5-HIAA	5-Hydroxyindole-3-acetic acid	54-16-0
TRP	Tryptophan	73-22-3
XA	Xanthurenic acid	59-00-7
ILA	Indole-3-lactic acid	1821-52-9
KYN	Kynurenine	343-65-7
MT	α-Methyltryptophan	153-91-3
NAS	N-Acetylserotonin	1210-83-9
5-HTP	5-Hydroxytryptophan	56-69-9
HK	3-Hydroxykynurenine	484-78-6
MEL	Melatonin	73-31-4
Trp-d3	Tryptophan-d3 (internal standard)	340257-61-6

Table S2. Instruments used for metabolite extraction

Equipment	Brand	Model
Refrigerated centrifuge	Hunan Xiangyi	H1650-W
Vortex mixer	Vortex Mixer	QL-866
Tissue grinder	Xinzhi	MB-96

Table S3. LC-MS/MS system

Instrument	Brand	Model
Ultra-high-performance liquid chromatograph	Waters	ACQUITY UPLC
Triple quadrupole mass spectrometer	AB Sciex	API 5000

1.2 Preparation of calibration standards

Appropriate amounts of each of the 30 tryptophan standards were weighed individually and dissolved in 50% (v/v) methanol to prepare individual stock solutions. Equal aliquots of the individual stocks were then combined to yield a mixed standard solution, which was serially diluted with 10% (v/v) methanol to prepare a twelve-point calibration curve for each analyte. The working concentration ranges spanned approximately 0.005-2500 ng/mL depending on the analyte. Both stock and working solutions were stored at 0 °C (Table 4).

Table S4. Standard curve concentration of tryptophan

Concentration unit: (ng/mL)												
Name	Cal1	Cal2	Cal3	Cal4	Cal5	Cal6	Cal7	Cal8	Cal9	Cal10	Cal11	Cal12
2-AP	50.000	25.000	12.500	6.250	3.130	1.560	0.781	0.391	0.195	0.0977	0.0488	0.0244
IND	2000.000	1000.000	500.000	250.000	125.000	62.500	31.300	15.600	7.810	3.910	1.950	0.977
NAm	125.000	62.500	31.300	15.600	7.810	3.910	1.950	0.977	0.488	0.244	0.122	0.061
Na	500.000	250.000	125.000	62.500	31.300	15.600	7.810	3.910	1.950	0.977	0.488	0.244
PA	1250.000	625.000	313.000	156.000	78.100	39.100	19.500	9.770	4.880	2.440	1.220	0.610
3MI	2500.000	1250.000	625.000	313.000	156.000	78.100	39.100	19.500	9.770	4.880	2.440	1.220
IAld	25.000	12.500	6.250	3.130	1.560	0.781	0.391	0.195	0.0977	0.0488	0.0244	0.0122
5-HAA	1000.000	500.000	250.000	125.000	62.500	31.300	15.600	7.810	3.910	1.950	0.977	0.488
3-HAA	250.000	125.000	62.500	31.300	15.600	7.810	3.910	1.950	0.977	0.488	0.244	0.122
TRM	50.000	25.000	12.500	6.250	3.130	1.560	0.781	0.391	0.195	0.0977	0.0488	0.0244
IEt	100.000	50.000	25.000	12.500	6.250	3.130	1.560	0.781	0.391	0.195	0.0977	0.0488
I3CA	200.000	100.000	50.000	25.000	12.500	6.250	3.130	1.560	0.781	0.391	0.195	0.0977
QA	5000.000	2500.000	1250.000	625.000	313.000	156.000	78.100	39.100	19.500	9.770	4.880	2.440
IAM	25.000	12.500	6.250	3.130	1.560	0.781	0.391	0.195	0.0977	0.0488	0.0244	0.0122
IAA	125.000	62.500	31.300	15.600	7.810	3.910	1.950	0.977	0.488	0.244	0.122	0.061
SER	50.000	25.000	12.500	6.250	3.130	1.560	0.781	0.391	0.195	0.0977	0.0488	0.0244
IArA	100.000	50.000	25.000	12.500	6.250	3.130	1.560	0.781	0.391	0.195	0.0977	0.0488
IPA	50.000	25.000	12.500	6.250	3.130	1.560	0.781	0.391	0.195	0.0977	0.0488	0.0244
KYNA	50.000	25.000	12.500	6.250	3.130	1.560	0.781	0.391	0.195	0.0977	0.0488	0.0244
MeOTA	25.000	12.500	6.250	3.130	1.560	0.781	0.391	0.195	0.0977	0.0488	0.0244	0.0122
5-HIAA	100.000	50.000	25.000	12.500	6.250	3.130	1.560	0.781	0.391	0.195	0.0977	0.0488
TRP	250.000	125.000	62.500	31.300	15.600	7.810	3.910	1.950	0.977	0.488	0.244	0.122
XA	100.000	50.000	25.000	12.500	6.250	3.130	1.560	0.781	0.391	0.195	0.0977	0.0488

ILA	250.000	125.000	62.500	31.300	15.600	7.810	3.910	1.950	0.977	0.488	0.244	0.122
KYN	125.000	62.500	31.300	15.600	7.810	3.910	1.950	0.977	0.488	0.244	0.122	0.061
MT	25.000	12.500	6.250	3.130	1.560	0.781	0.391	0.195	0.0977	0.0488	0.0244	0.0122
NAS	10.000	5.000	2.500	1.250	0.625	0.313	0.156	0.0781	0.0391	0.0195	0.00977	0.00488
5-HTP	100.000	50.000	25.000	12.500	6.250	3.130	1.560	0.781	0.391	0.195	0.0977	0.0488
HK	200	100	50	25	12.5	6.25	3.13	1.56	0.781	0.391	0.195	0.0977
MEL	50	25	12.5	6.25	3.13	1.56	0.781	0.391	0.195	0.0977	0.0488	0.0244

1.3 Metabolite extraction

Serum samples: An aliquot of 50 μL serum was transferred into a 2 mL EP tube. After adding 100 μL of 80% methanol aqueous solution, 900 μL of 10% methanol aqueous solution was subsequently added. The mixture was vortexed for 30 s and centrifuged at 12,000 rpm for 5 min at 4 °C. A 100 μL aliquot of the supernatant was collected and mixed with 100 μL of 10 ppb internal standard solution, followed by vortexing for 30 s. The supernatant was then filtered through a 0.22 μm membrane, and the filtrate was transferred into an autosampler vial for LC-MS/MS analysis.

Tissue/content samples: Approximately 50 mg of sample was accurately weighed into a 2 mL EP tube with one stainless steel bead. After adding 100 μL of 80% methanol aqueous solution, the sample was homogenized using a high-throughput tissue grinder for 60 s. Subsequently, 900 μL of 10% methanol aqueous solution was added, and the homogenization was continued for another 120 s. The mixture was then centrifuged at 12,000 rpm for 10 min at 4 °C. A 100 μL aliquot of the supernatant was diluted with 400 μL of 10% methanol aqueous solution and vortexed for 30 s. Thereafter, 100 μL of the diluted sample was mixed with 100 μL of 10 ppb internal standard solution, followed by vortexing for 30 s. The supernatant was filtered through a 0.22 μm membrane, and the filtrate was transferred into an autosampler vial for LC-MS/MS analysis.

1.4 Quantification

The concentration of each analyte in the injected solution (C, ng/mL) was obtained from the corresponding calibration curve using the peak-area ratio against the internal standard. The content in the original sample was calculated as:

$$\text{Content } (\mu g/g) = C \times 1 \times 2 \times 5 / \text{sample weight (mg or } \mu L)$$

1.5 Chromatographic conditions

Chromatographic separation was performed on an ACQUITY UPLC® HSS T3 column (2.1 × 150 mm, 1.8 µm; Waters, Milford, MA, USA). The injection volume was 5 µL and the column temperature was maintained at 40 °C. The mobile phase consisted of 0.1% (v/v) formic acid in water (A) and 0.1% (v/v) formic acid in methanol (B), delivered at a flow rate of 0.3 mL/min. The gradient elution program was as follows: 0-2 min, 1% B; 2-3 min, 1%-30% B; 3-3.5 min, 30% B; 4.5-8 min, 30%-50% B; 8-10 min, 50%-95% B; 10-11 min, 95% B; 11-17 min, 95%-1% B.

1.6 Mass spectrometric conditions

Mass spectrometric detection was carried out on an AB Sciex API 5000 triple quadrupole mass spectrometer equipped with an electrospray ionization (ESI) source operated in positive-ion mode. The ion source temperature was set to 500 °C and the ion spray voltage to 5500 V. Collision gas (CAD), curtain gas (CUR), nebulizer gas (GS1) and auxiliary gas (GS2) were set at 6, 30, 50 and 50 psi, respectively. Analytes were quantified in multiple reaction monitoring (MRM) mode with scheduled retention-time windows. The precursor/product ion transitions and compound-dependent parameters (Declustering Potential, DP; Entrance Potential, EP; Collision Energy, CE; Collision cell Exit Potential, CXP) are summarized in Table 5.

Table S5. MRM transitions and mass spectrometric parameters for the quantified analytes

No.	Abbrev.	Q1 (m/z)	Q3 (m/z)	DP (V)	EP (V)	CE (V)	CXP (V)
1	2-AP	110.053	65.000	101	10	31	26
2	IND	118.273	91.000	66	10	31	6
3	NAm	123.057	80.000	76	10	29	6
4	NA	124.021	79.900	66	10	29	6
5	PA	124.091	78.000	36	10	29	6
6	3MI	132.071	117.000	66	10	29	10
7	IAld	146.120	117.900	71	10	21	10
8	5-HAA	154.082	136.100	116	10	15	14
9	3-HAA	154.090	135.900	116	10	17	18
10	TRM	161.053	144.000	31	10	13	12
11	IEt	162.057	143.900	106	10	19	14
12	I3CA	162.069	117.900	66	10	17	8
13	QA	168.046	77.800	46	10	31	6
14	IAM	175.054	130.000	66	10	19	12
15	IAA	176.087	130.000	56	10	19	12
16	SER	177.122	160.000	46	10	15	14
17	IArA	188.023	170.100	61	10	19	4
18	IPA	189.993	130.100	66	10	19	12

No.	Abbrev.	Q1 (m/z)	Q3 (m/z)	DP (V)	EP (V)	CE (V)	CXP (V)
19	KYNA	190.031	162.200	71	10	23	4
20	MeOTA	191.252	174.300	51	10	17	14
21	5-HIAA	192.048	146.000	56	10	21	14
22	TRP	205.037	187.900	41	10	15	18
23	XA	206.054	178.200	71	10	25	4
24	ILA	206.081	118.000	71	10	31	10
25	KYN	209.089	93.900	46	10	21	8
26	MT	219.102	201.900	51	10	15	6
27	NAS	219.108	160.100	76	10	21	16
28	5-HTP	221.106	204.200	46	10	17	6
29	HK	225.088	208.000	61	10	13	6
30	MEL	233.159	174.100	120	10	50	15
31	Trp-d3	208.185	191.000	41	10	15	26

1.7 Method validation

1.7.1 Calibration curves and limits of quantification

Each working standard solution was analyzed by LC–MS. Linearity was evaluated by constructing calibration curves with analyte concentration on the x-axis and the corresponding peak-area ratio on the y-axis. The linear regression equations for the individual tryptophan-related analytes are provided in the analytical report, and all correlation coefficients (*r*) exceeded 0.99. The limit of quantification (LOQ) and limit of detection (LOD) were determined using the signal-to-noise (S/N) method by comparing analyte signals in low-concentration samples with the background noise in blank samples. The LOQ and LOD were defined as the concentrations corresponding to S/N ratios of 10:1 and 3:1, respectively.

1.7.2 Precision

Working standard solutions at different concentrations were injected five consecutive times within the same analytical batch (n = 5). Within-batch precision was calculated and expressed as the relative standard deviation (RSD). Five batches of standard samples at each concentration level were prepared and analyzed at different times (n = 5). Between-batch precision was calculated from the five batch means at each concentration level and expressed as the RSD. Overall precision was calculated using all measurements from the five batches at each concentration level and expressed as the RSD. The RSD values for within-batch, between-

batch, and overall precision were all below 15%, indicating good instrumental precision. Detailed results are presented in Table 6.

1.7.3 Repeatability

Six replicate sample preparations (n = 6) were performed according to the procedure described under “Sample Pretreatment.” Repeatability was assessed as the RSD of the measured concentrations. Detailed results are presented in Table 6.

1.7.4 Stability

Working standard solutions at different concentrations were stored in the autosampler for 1, 2, 4, 5, and 6 days. Fresh calibration standards were prepared daily to construct calibration curves. Stability was expressed as the measured concentration as a percentage of the nominal concentration. Although a few analytes showed values outside the 85-115% acceptance range, the remaining analytes exhibited acceptable stability for up to 6 days. Detailed results are presented in Table 6.

1.7.5 Matrix effect

Samples were homogenized with 100 μL of 80% methanol at 50 Hz for 1 min. Subsequently, 900 μL of 10% methanol was added, followed by further homogenization for 2 min and centrifugation at 12,000 rpm for 5 min. A 50-μL aliquot of the supernatant was mixed with 50 μL of either 10% methanol or a mixed standard solution at the designated concentration, together with 100 μL of internal standard solution (10 ppb Trp-d3 prepared in 10% methanol). The mixture was vortexed for 10 s and centrifuged at 12,000 rpm for 5 min, and the resulting supernatant was subjected to LC-MS analysis.

QC samples at each concentration level were processed in six replicates (n = 6), and matrix effects were evaluated on the same day. Matrix effects were calculated on a mass basis using the following equation: Matrix effect (%) = [(spiked response - blank response) / amount actually added] × 100. The results are presented in Table 7. Matrix effects for some analytes fell outside the predefined acceptance range of 85%–115%, whereas those for the remaining analytes met this criterion ().

1.7.6 Recovery

Samples were homogenized with 100 μL of 80% methanol at 50 Hz for 1 min. Subsequently, 900 μL of either 10% methanol or a mixed standard solution at the designated

concentration was added, followed by further homogenization for 2 min and centrifugation at 12,000 rpm for 5 min. A 100-μL aliquot of the supernatant was mixed with 100 μL of internal standard solution (10 ppb Trp-d3 prepared in 10% methanol). The mixture was vortexed for 10 s and centrifuged at 12,000 rpm for 5 min, and the resulting supernatant was subjected to LC-MS analysis.

QC samples at each concentration level were processed in six replicates (n = 6), and recovery was evaluated on the same day. Recovery was calculated on a mass basis using the following equation: Recovery (%) = [(spiked response - blank response) / theoretical amount added] × 100. The results are presented in Table 7. Recoveries for some analytes fell outside the predefined acceptance range of 85%-115%, whereas those for the remaining analytes met this criterion.

1.7.7 Quality control

QC injections were interspersed among sample injections within each analytical batch. Method stability was assessed using the RSD of peak areas across QC injections (Table 8).

1.9 Reference

[1] Chen G-Y, Zhong W, et al. Simultaneous determination of tryptophan and its 31 catabolites in mouse tissues by polarity switching UHPLC-SRM-MS[J]. Anal Chim Acta, 2018, 1037: 200–210.

Table S6. Intra-batch, inter-batch and total precision, repeatability, and stability

No.	Abbrev.	Conc. (ng/mL)	Intra-batch RSD (%)	Inter-batch RSD (%)	Total RSD (%)	Repeatability RSD (%)	Day 1 (%)	Day 2 (%)	Day 4 (%)	Day 5 (%)	Day 6 (%)
1	2-AP	0.77	7.51	2.65	5.46	3.97	92.58	90.32	85.00	90.02	91.30
		2.8	7.17	4.87	5.93		86.78	85.20	85.48	86.64	85.90
		11.38	6.23	5.91	6.57		85.22	96.00	89.52	95.08	103.78
2	IND	28.75	5.85	4.50	6.54	3.75	88.74	86.68	92.54	90.88	89.46
		119.89	7.27	8.40	8.58		88.44	92.54	94.30	87.46	87.36
		527	3.24	6.28	6.57		89.20	113.60	109.40	100.36	105.44
3	NAm	1.84	8.75	5.90	8.29	12.81	98.64	89.70	92.16	107.48	99.70
		7.29	4.67	6.53	7.72		86.50	113.40	89.48	95.86	110.20
		28	7.89	11.27	11.48		78.08	111.80	86.50	96.00	104.16
4	NA	7.95	3.44	5.77	7.36	5.79	113.20	107.64	103.48	105.44	99.92
		31.97	5.40	4.77	5.45		97.62	98.66	95.92	104.80	104.94
		115.7	8.22	5.84	7.33		81.82	94.84	89.44	108.40	98.30
5	PA	21.26	4.11	2.01	3.78	6.31	103.80	110.40	90.30	101.96	100.38
		80.97	4.85	5.63	5.97		91.86	100.30	97.92	101.06	93.90
		284.37	2.98	3.74	4.52		85.16	94.68	91.94	101.52	98.42
6	3MI	34.93	6.21	5.54	7.18	8.33	88.98	88.84	89.76	88.78	92.14
		146.2	6.80	8.02	8.22		87.72	94.96	86.98	88.34	89.68
		655.03	5.47	2.20	3.53		93.34	109.40	105.80	107.80	110.80
7	IAld	0.38	3.32	6.31	6.66	8.10	88.24	86.60	107.32	90.26	108.80
		1.53	5.80	4.43	5.31		90.56	85.80	96.20	86.58	102.04
		5.83	5.73	6.05	6.75		85.44	90.94	92.94	87.88	104.96
8	5-HAA	16.1	9.83	4.28	6.48	7.69	94.58	104.60	86.34	89.60	90.74
		60.55	4.79	6.08	7.41		86.16	97.60	90.40	90.72	89.98
		225.77	8.72	2.88	5.15		85.58	94.56	89.06	94.36	103.34
9	3-HAA	4.15	3.02	2.47	3.58	5.07	105.80	110.40	88.64	87.98	97.52
		15.43	9.06	7.63	8.43		93.94	97.54	90.70	86.44	86.38
		57.5	8.47	6.65	7.71		86.98	102.14	91.76	90.22	95.16
10	TRM	0.79	4.02	4.99	5.45	7.09	94.32	88.10	92.12	87.94	101.60
		3.04	4.80	3.50	4.54		90.88	86.42	95.58	88.60	91.90
		11.78	6.38	2.23	4.37		86.66	90.74	93.76	95.96	99.50
11	IEt	1.56	5.83	3.68	5.15	3.69	93.40	88.72	99.24	93.94	107.60
		6.05	6.99	3.82	5.20		90.76	85.88	92.88	89.62	97.34
		22.67	4.51	3.24	4.76		89.68	88.98	90.94	89.74	99.52
12	I3CA	3.27	5.85	3.96	5.05	9.40	101.16	93.80	95.64	104.60	109.00
		12.65	4.13	5.39	5.60		97.42	88.02	89.52	96.88	102.98
		45.63	5.31	5.39	5.93		85.32	88.50	85.22	94.02	99.14

No.	Abbrev.	Conc. (ng/mL)	Intra-batch RSD (%)	Inter-batch RSD (%)	Total RSD (%)	Repeatability RSD (%)	Day 1 (%)	Day 2 (%)	Day 4 (%)	Day 5 (%)	Day 6 (%)
13	QA	78.3	4.03	7.44	8.69	2.82	88.70	90.00	105.80	104.64	95.70
		318	9.83	6.39	8.97		89.48	92.64	102.04	102.06	102.24
		1275.67	5.65	3.13	6.42		86.18	108.80	98.90	103.34	95.00
14	IAM	0.4	3.30	3.74	5.98	4.27	94.04	94.26	100.14	109.00	102.98
		1.56	8.41	6.30	7.29		93.14	88.40	92.10	103.12	106.42
		5.78	5.45	8.99	9.15		86.16	92.08	86.46	98.96	100.42
15	IAA	2.03	4.22	2.64	3.76	6.82	98.54	100.92	99.08	95.06	113.60
		7.72	6.39	4.99	5.76		94.60	96.22	92.02	87.12	102.50
		27.55	3.92	6.04	6.15		85.70	94.64	85.04	85.04	98.28
16	SER	0.75	4.35	10.63	10.26	7.60	88.24	85.82	88.18	86.42	99.88
		2.79	10.06	9.78	10.43		86.22	88.16	94.92	85.70	92.38
		11.15	6.71	5.39	6.30		86.36	88.32	94.18	89.68	97.40
17	IArA	1.74	3.59	5.25	5.83	7.60	110.20	104.00	109.60	111.80	109
		6.65	5.05	2.89	4.15		101.80	105.20	97.62	102.40	101.14
		22.26	2.96	5.42	5.78		86.06	97.10	85.34	86.78	89.82
18	IPA	0.83	3.85	2.01	3.23	12.74	103.20	102.70	102.22	105.00	109.20
		3.26	2.77	4.49	4.75		99.52	100.08	94.38	97.58	102.22
		11.37	5.08	5.61	5.99		86.74	100.28	86.50	87.84	92.68
19	KYNA	0.79	7.33	5.36	8.25	4.93	93.36	96.94	101.42	103.60	94.16
		3.26	4.10	2.88	3.96		100.46	96.34	96.60	106.38	99.54
		12.33	2.80	2.32	4.50		91.02	105.60	96.14	111.20	110.20
20	MeOTA	0.39	6.25	5.02	5.80	8.88	93.28	86.80	86.90	85.70	97.14
		1.49	6.83	3.86	5.36		90.72	85.00	90.80	87.48	90.88
		6.03	6.35	1.52	3.88		88.88	96.00	94.72	98.72	97.58
21	5-HIAA	1.59	7.80	4.33	5.87	8.02	100.70	96.34	104.44	92.26	104.00
		6.2	6.30	4.41	5.76		96.50	90.36	99.64	90.20	93.84
		23.27	6.57	2.78	5.10		91.12	93.98	95.92	97.34	100.82
22	TRP	3.97	9.63	5.88	6.79	12.59	107.20	107.40	103.28	113.00	91.06
		16.23	5.27	2.82	4.40		108.60	99.84	96.34	97.32	98.24
		59.62	3.53	5.53	6.10		94.16	100.20	90.38	94.08	107.40
23	XA	1.58	7.21	2.49	5.22	2.57	107.80	89.70	94.70	96.54	103.66
		6.18	6.58	3.60	5.62		99.58	85.88	90.62	91.08	97.20
		23.44	5.81	5.76	7.05		95.36	88.98	91.50	93.54	100.24
24	ILA	4.04	4.30	2.30	3.58	8.38	105.40	96.40	100.20	100.40	110.60
		15.73	4.36	1.70	3.20		101.60	90.74	95.28	92.94	99.92
		57.79	3.57	2.90	4.06		93.06	92.52	90.92	94.20	101.36
25	KYN	2.01	3.50	4.01	4.62	7.47	102.16	95.72	95.98	97.70	105.80
		7.66	6.95	2.06	4.52		98.76	88.36	89.68	88.80	93.64

No.	Abbrev.	Conc. (ng/mL)	Intra-batch RSD (%)	Inter-batch RSD (%)	Total RSD (%)	Repeatability RSD (%)	Day 1 (%)	Day 2 (%)	Day 4 (%)	Day 5 (%)	Day 6 (%)
		28.28	6.57	1.62	5.23		92.12	89.66	87.78	93.22	95.62
26	MT	0.39	8.81	3.32	6.30	6.65	97.96	91.50	98.60	99.94	98.58
		1.54	9.83	2.64	6.55		100.14	87.06	100.46	97.16	93.56
		5.89	8.13	3.20	6.00		95.02	95.02	98.00	101.38	99.86
27	NAS	0.16	6.39	3.28	4.96	7.68	100.08	89.06	98.18	99.48	104.20
		0.61	8.50	2.80	4.96		101.00	85.44	92.70	89.30	92.30
		2.32	6.35	3.01	5.66		94.42	91.98	90.92	94.08	95.32
28	5-HTP	1.58	6.29	2.11	4.69	7.55	98.92	88.18	93.48	98.78	104.14
		6.07	3.52	1.38	4.14		96.82	86.30	90.28	93.20	95.68
		22.89	5.35	2.96	4.82		93.28	91.06	86.02	94.64	91.98
29	HK	3.12	9.80	3.73	6.54	18.61	100.84	88.56	96.62	93.52	102.06
		12.1	10.27	1.90	5.32		100.18	85.78	92.92	92.62	93.20
		46.09	5.16	2.97	5.71		91.78	89.42	87.34	95.06	93.16
30	MEL	0.8	3.93	3.90	5.95	13.81	100.80	88.18	98.18	96.88	107.80
		3.1	4.05	2.90	4.15		96.18	85.56	89.10	90.68	98.04
		11.64	4.21	4.53	5.47		89.96	88.32	87.20	91.24	101.20

Table S7 Matrix effects and recovery

No.	Abbrev.	Actual amount added (ng)	Matrix effect (%)	Matrix-effect RSD (%)	Theoretical amount added (ng)	Recovery (%)	Recovery RSD (%)
1	2-AP	0.14	106.57	9.94	1.41	94.41	7.22
		0.55	112.64	5.85	5.63	87.33	3.04
		2.61	89.51	6.05	22.50	94.37	5.31
2	IND	5.69	101.88	14.38	56.25	94.58	2.81
		21.82	107.05	7.28	225.00	94.34	11.94
		97.30	107.91	9.88	900.00	105.49	13.71
3	NAm	0.39	88.40	10.43	3.52	101.65	8.77
		1.34	93.13	9.57	14.06	86.35	3.21
		5.35	88.67	2.49	56.25	85.55	5.13
4	NA	1.65	102.04	10.17	14.06	97.29	12.47
		5.68	103.37	10.48	56.25	91.82	14.03
		21.60	103.09	6.92	225.00	90.59	5.53
5	PA	3.98	92.04	9.47	35.16	94.14	5.61
		13.43	99.38	6.93	140.63	93.73	3.46
		47.10	95.82	8.75	562.50	86.12	5.48
6	3MI	7.46	110.82	8.06	70.31	105.24	4.27
		29.87	114.73	5.91	281.25	109.16	2.09

No.	Abbrev.	Actual amount added (ng)	Matrix effect (%)	Matrix-effect RSD (%)	Theoretical amount added (ng)	Recovery (%)	Recovery RSD (%)
		141.87	107.92	7.19	1125.00	110.22	5.40
7	IAld	0.08	88.70	10.83	0.70	90.52	5.52
		0.28	105.26	10.75	2.81	94.11	14.34
		1.36	92.41	7.05	11.25	96.42	14.81
8	5-HAA	2.89	70.58	12.07	28.13	88.06	4.52
		11.40	81.78	11.15	112.50	65.69	6.61
		41.20	87.62	5.97	450.00	72.00	8.29
9	3-HAA	0.75	111.21	7.15	7.03	109.23	5.42
		2.82	116.31	3.51	28.13	109.39	7.03
		11.99	104.72	9.66	112.50	101.93	6.22
10	TRM	0.15	98.19	14.68	1.41	92.73	13.09
		0.57	114.53	7.06	5.63	85.48	14.85
		2.66	97.65	9.10	22.50	89.06	9.75
11	IEt	0.30	106.48	12.38	2.81	101.24	12.61
		1.16	113.23	9.33	11.25	106.57	6.50
		5.31	88.54	8.83	45.00	108.39	5.35
12	I3CA	0.67	100.94	12.67	5.63	94.46	12.53
		2.41	108.22	4.89	22.50	111.59	7.34
		10.07	95.58	7.03	90.00	109.17	5.50
13	QA	14.98	95.11	12.82	140.63	103.38	9.32
		55.93	102.86	8.56	562.50	108.50	6.45
		222.00	95.99	8.58	2250.00	108.44	8.04
14	IAM	0.09	111.35	9.22	0.70	101.11	9.26
		0.32	114.85	0.95	2.81	113.99	4.79
		1.43	103.18	6.07	11.25	114.91	5.56
15	IAA	0.45	90.62	6.97	3.52	67.74	12.75
		1.59	94.30	7.08	14.06	110.00	13.70
		5.97	97.39	7.40	56.25	89.20	11.27
16	SER	0.14	113.02	9.86	1.41	85.36	7.26
		0.58	114.85	4.54	5.63	72.69	10.38
		2.48	106.94	9.76	22.50	85.09	3.99
17	IArA	0.34	102.95	12.28	2.81	111.56	6.99
		1.17	113.75	1.85	11.25	114.74	3.37
		4.70	109.02	6.01	45.00	114.85	3.95
18	IPA	0.17	93.19	26.18	1.41	99.61	12.02
		0.61	107.41	5.31	5.63	97.29	10.56
		2.56	98.03	6.77	22.50	103.47	5.49
19	KYNA	0.17	98.08	9.81	1.41	89.71	7.86
		0.60	104.12	11.85	5.63	101.72	9.76

No.	Abbrev.	Actual amount added (ng)	Matrix effect (%)	Matrix-effect RSD (%)	Theoretical amount added (ng)	Recovery (%)	Recovery RSD (%)
		2.23	91.60	10.25	22.50	88.84	12.91
20	MeOTA	0.08	112.05	8.73	0.70	88.37	4.58
		0.31	114.32	8.14	2.81	88.06	2.77
		1.44	101.39	8.86	11.25	93.96	7.34
21	5-HIAA	0.31	96.31	13.99	2.81	98.00	13.50
		1.23	88.01	5.94	11.25	94.57	7.54
		4.38	87.07	9.47	45.00	87.84	10.68
22	TRP	0.86	97.99	14.49	7.03	98.28	11.42
		2.81	107.04	11.11	28.13	97.90	14.42
		10.82	89.95	11.14	112.50	85.75	14.82
23	XA	0.33	98.63	13.32	2.81	102.60	14.74
		1.19	86.33	5.60	11.25	97.35	7.05
		3.99	86.04	8.53	45.00	72.86	11.27
24	ILA	0.88	89.45	10.15	7.03	92.33	12.53
		3.18	89.68	8.08	28.13	104.64	8.03
		12.04	89.97	5.26	112.50	105.32	5.77
25	KYN	0.41	91.54	8.65	3.52	89.10	7.50
		1.55	99.91	6.08	14.06	91.76	5.46
		6.07	95.94	6.40	56.25	95.54	6.54
26	MT	0.09	104.41	10.24	0.70	107.66	14.69
		0.33	106.45	5.98	2.81	91.97	4.58
		1.39	96.30	7.70	11.25	99.44	6.30
27	NAS	0.03	100.52	14.77	0.28	90.03	5.07
		0.12	113.92	4.44	1.13	94.27	12.61
		0.57	97.67	5.71	4.50	108.05	7.15
28	5-HTP	0.34	107.26	8.59	2.81	107.02	3.48
		1.17	114.92	6.11	11.25	103.08	4.25
		5.35	102.62	10.66	45.00	108.44	6.16
29	HK	0.66	100.05	10.21	5.63	98.84	4.08
		2.42	111.14	6.57	22.50	99.70	3.06
		10.49	102.86	4.94	90.00	105.93	6.66
30	MEL	0.15	99.32	11.12	1.41	94.32	11.67
		0.56	114.76	6.21	5.63	104.40	6.98
		2.67	92.92	8.07	22.50	113.05	7.66

Table S8. QC stability in colonic and ileum digesta and serum

Analyte	QC RSD (%) Colonic digesta	QC RSD (%) Serum	QC RSD (%) Ileum digesta
2-AP	ND	ND	ND
IND	10.21	ND	8.460
NAm	ND	2.40	9.390
NA	5.34	ND	2.180
PA	4.14	ND	ND
3MI	2.98	ND	ND
IAld	3.22	12.04	9.870
5-HAA	7.49	ND	ND
3-HAA	0.86	ND	1.960
TRM	5.12	ND	6.740
IEt	4.31	ND	9.610
I3CA	5.91	ND	0.120
QA	ND	5.51	2.330
IAM	4.62	ND	3.100
IAA	4.98	12.73	6.090
SER	5.05	12.85	ND
IArA	ND	ND	ND
IPA	3.54	6.49	ND
KYNA	3.36	12.67	2.250
MeOTA	ND	ND	ND
5-HIAA	ND	ND	ND
TRP	4.94	5.15	3.100
XA	3.61	ND	3.370
ILA	7.26	10.60	1.450
KYN	12.23	9.51	1.880
MT	ND	ND	ND
NAS	10.98	ND	4.990
5-HTP	10.44	11.05	ND
HK	ND	12.18	ND
MEL	ND	ND	ND
